# Without Contact Resistance, Proteins in Thin‐Film Solid‐State Junctions Can Be Efficient Electronic Conducting Materials

**DOI:** 10.1002/adma.202507654

**Published:** 2025-09-19

**Authors:** Sudipta Bera, Ayelet Vilan, Sourav Das, Israel Pecht, David Ehre, Mordechai Sheves, David Cahen

**Affiliations:** ^1^ Department of Molecular Chemistry and Materials Science Weizmann Institute of Science Rehovot 7610001 Israel; ^2^ Department of Chemical Research Support Weizmann Institute of Science Rehovot 7610001 Israel; ^3^ Department of Immunology and Regenerative Biology Weizmann Institute of Science Rehovot 7610001 Israel

**Keywords:** contact resistance, impedance, metal–metal junctions, micropore devices, protein junctions

## Abstract

The solid‐state protein junctions have shown efficient electron transport over a few tens of nanometer lengthscale. This work demonstrates, how the contact resistance ($\hat{R_{C}}$) of a solid‐state protein junctions, treated as a contact‐limited process, which can be extracted quantitatively from the measured junction resistance (*R*_P_) by using the extrapolated zero‐length resistance and series resistance (*R*_S_). Alternating current (impedance spectroscopy) and direct current measurements are used to examine charge transport in junctions of human serum albumin (HSA) and bacteriorhodopsin (bR) films with varying thicknesses. Three contact configurations, Si–Au, Au–eutectic gallium indium (EGaIn), and, in a micropore device (MpD), Au–Pd, are compared. While Si–Au and Au–EGaIn junctions exhibit substantial $\hat{R_{C}}$ that are ascribed to interfacial oxides and electrostatic protein–electrode interactions, MpD effectively eliminates $\hat{R_{C}}$, enabling measuring the intrinsic electron transport across HSA and bR films. The exponential length‐dependence of *R*_P_ shows a transport decay constant (*β*) that varies with interfacial conditions, underscoring the role of contact engineering. By minimizing $\hat{R_{C}}$, exceptionally low *β* values (≈0.7–1.1 nm^−1^) are found, proving that, indeed, proteins can have outstanding charge transport efficiencies.

## Graphical abstract

It is demonstrated that contact resistance is effectively eliminated in two‐probe micropore device metal/protein/metal bioelectronic junctions. These results, using two different protein types in these junctions, show that, as ultrathin (<40 nm) films, proteins are an even more efficient electronic transport medium than previously thought, when the contact resistance is still included in measurements.

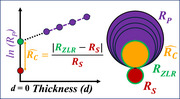

## 1 Introduction

Recent reports on electron transport across protein films, with transport distances spanning tens of nanometers,^[^ ^1^, ^2^, ^3^, ^4^, ^5^ ^]^ suggest that proteins in solid‐state junctions serve as a surprisingly efficient electron transport medium. The fact that these distances far exceed expectations for a medium with a significant saturated organic component, coupled with the lack of temperature dependence,^[^ ^2^, ^6^ ^]^ challenges our understanding of existing transport mechanisms. A theoretical cascade model, proposed by Papp et al.^[^ ^7^ ^]^ predicts that a single contact dominates the transport via nanometer‐long proteins. In this model, the more resistive contact, rather than the protein itself, largely governs overall transport efficiency.^[^ ^2^ ^]^ Futera et al.,^[^ ^8^ ^]^ using density functional theory and molecular dynamics, it was shown that small (<3 nm) tetraheme protein junctions can function as one complete electrode–protein–electrode composite.

Testing a model for electron transport through proteins requires experimental determination of the intrinsic transport properties of the protein in the junctions. To that end, junctions should be such that the contribution of the contacts to the measured transport is minimal. This challenge is not unique to protein junctions; in molecular electronics, interfacial effects often overshadow intrinsic molecular properties, defining characteristics of the field since its inception. In this regard, molecular junctions are not too different from other junctions, as demonstrated in textbooks on metal–metal contacts (see, e.g., ref. [9]). Though there is a basic difference between the studied molecular junctions,^[^ ^10^, ^11^, ^12^, ^13^ ^]^ which mostly have junction widths of 1 to a few nanometers, and the protein systems investigated in this work and other recent studies,^[^ ^2^, ^14^ ^]^ which involve protein multilayer junctions spanning several tens of nanometers and lie well beyond the tunneling length scale, the assumptions and predictions based on quantum mechanical tunneling do not apply to protein multilayer junctions. We stress that the present work focuses specifically on long (mostly ⪆10 nm) protein junctions, which exclude QM tunneling as a transport mechanism. As shown before,^[^ ^2^, ^15^ ^]^ its lack of temperature‐dependence^[^ ^14^ ^]^ also excludes hopping, i.e., this work. also further emphasizes our present lack of understanding of mechanisms of long‐range electron transport across common (rather than special, e.g., multiheme,^[^ ^16^ ^]^ Ni–thiophene^[^ ^17^ ^]^) proteins.

A molecular junction can be conceptually divided into three key components: the two electrode–molecule contact interfaces and the molecular layer between them. Salomon et al.^[^ ^18^ ^]^ demonstrated that electron transmission across molecules is primarily governed by the molecule–electrode interfaces and the intrinsic properties of the electrode materials. Indeed, as Hipps noted for DNA junctions, molecule–contact interfaces dictate conductivity, leading to reports of insulating, semiconducting, conducting, and even superconducting behaviors.^[^ ^19^ ^]^

Achieving control over interfaces or contacts is challenging both conceptually and technically. How contacts are established can often have a more significant effect on molecular junction measurements than the molecules themselves. Even minor variations in a contact formation protocol can lead to substantial changes in device characteristics.^[^ ^20^ ^]^ The nature of the contacts determines if any material mixing across the interface can occur or if only electronic charge carriers will cross. Here, nature of contacts includes surface roughness (and, consequently, interface roughness), defect formation, activation or mitigation, and, in the case of soft materials, flexibility.^[^ ^21^, ^22^ ^]^ Therefore, to accurately assess the intrinsic charge transport capabilities of molecular junctions, contact effects should be first quantified and then minimized. This conclusion applies also to protein‐based junctions.

**Scheme 1:**
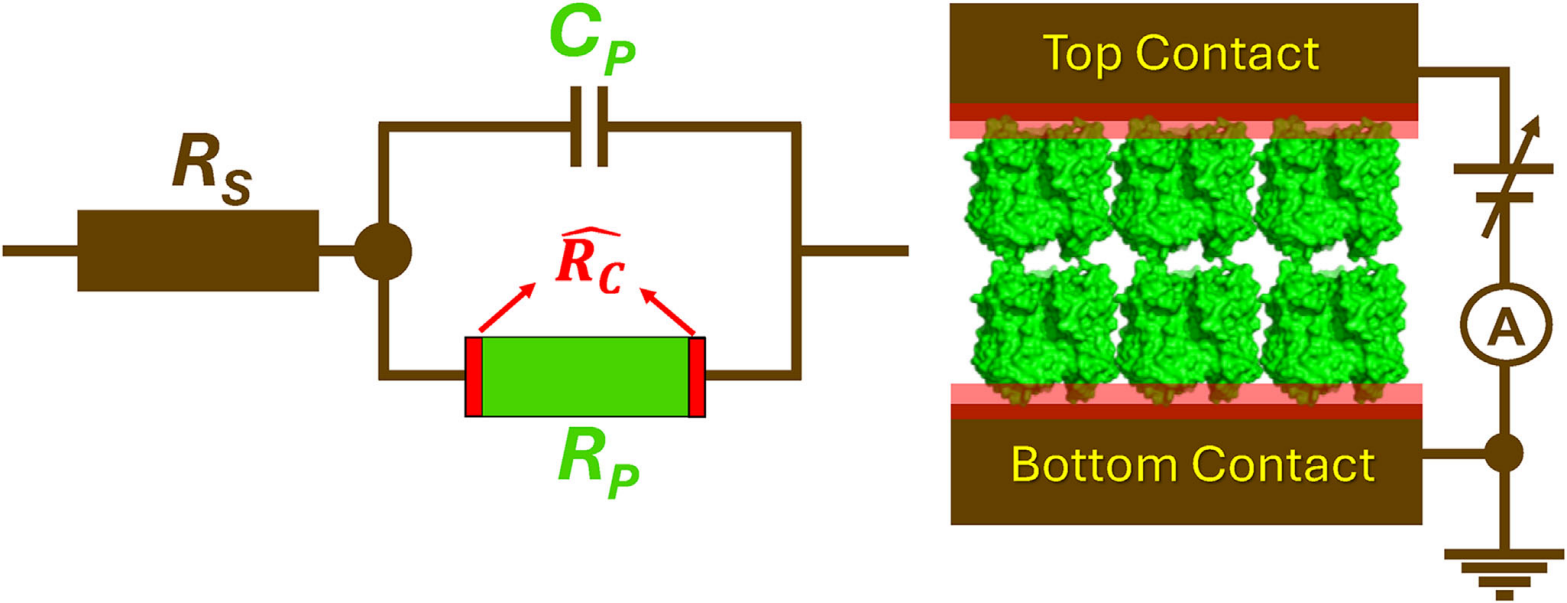
Left: equivalent circuit of solid‐state protein‐based devices, irrespective of protein type and junction configurations; series resistance (*R*_S_), protein resistance (*R*_P_), and protein capacitance (*C*_P_). Specific color coding in figure on the left represents different circuit components: green—protein part, dark brown— whole circuit with terminal leads and external wire connection without protein, and red—protein/electrode interface regions that contribute to contact resistance ($\hat{R_{C})}$. In practice, the value that is measured as *R*_P_ includes $\hat{R_{C}}$. Right: schematic cross‐section of the junction with the proteins (green) and the interface regions on both sides of the protein/electrode contact interfaces (light and darker red‐brown).

In this study the concept of $\hat{R_{C}}$ is used to account for the effects of contacts and their interfaces with proteins. Here, $\hat{R_{C}}$ is not in the strict sense of a resistor; rather, it represents the contact‐limited process for the protein‐based devices. Typically, $\hat{R_{C}}$ is mitigated using four‐probe measurements.^[^ ^23^ ^]^ However, for single‐molecule or ultrathin film systems, implementing a four‐probe setup would require electrodes spaced only a few nanometers apart, without any crosstalk, an approach that remains technically unfeasible. As a result, studies of molecular junctions have employed two different strategies for evaluating the different contributions to the junction resistance. One approach is the concept of zero‐length resistance (*R*_ZLR_), which is obtained by measuring the total resistance across the identical junctions, except for sample thickness (i.e., electrode‐to‐electrode separation), and exponentially extrapolating it to zero sample length.^[^ ^24^, ^25^ ^]^ *R*_ZLR_ can be derived from length‐dependent electron transport measurements of either direct current (DC) or alternating current (AC) measurements. The other approach uses impedance measurements (Section S1 in the Supporting Information) to separate the resistance at short‐circuit, i.e., if the two electrodes are in direct contact, without a sample between them, which is taken to give the series resistance (*R*_S_), and the junction (protein) resistance (*R*_P_, in parallel with a capacitor). We propose here that the combination of these two approaches reveals the contribution of the contact to the junction resistance. **Scheme** **1** presents individual circuit element^[^ ^2^, ^26^ ^]^ details, including $\hat{R_{C}}$ as part of *R*_P_. While formally the actual deviation between *R*_ZLR_ and *R*_S_​ defines contact resistance, because *R*_S_​ will normally be very small compared to the other resistances in the circuit, $\hat{R_{C}}$ ​will be dominated by *R*_ZLR_, which makes comparing $\hat{R_{C}}$ ​values between junction configurations problematic. To ensure scalability and meaningful comparison, here the contact resistance is reformulated in terms of dimensionless measure (represented as $\hat{R_{C}}$) that reflects the difference between *R*_ZLR_ and *R*_S_ relative to *R*_S_​, as follows

(1) $$\hat{R_{C}}=\frac{|R_{ZLR}\;-\;R_{S}|}{R_{S}}$$

Engelkes et al.^[^ ^25^ ^]^ used the *R*_ZLR_ directly as the $\hat{R_{C}}$ for metal/insulator/metal junctions in a conductive atomic force microscopy configuration. Notably, *R*_ZLR_ varies with contact type;^[^ ^24^ ^]^ it is strongly influenced by the metal electrode's work function,^[^ ^25^ ^]^ emphasizing the difference between “contact‐limited” and “contact‐influenced” transport. The latter includes the dependence of transport rates on contact details, such as the density of electronic states and their coupling strength to the medium. “Contact‐limited” refers to cases where the injection of carriers across the interface limits the net transport. Admittedly, it is not trivial to separate between these two aspects. Within the above‐suggested definition of “contact resistance,” cases where $\hat{R_{C}}\;$→ 0 implies that the net transport is not “contact‐limited.”

Also, the (short‐circuit) *R*_S_ can be evaluated by either AC or DC measurements. The AC‐impedance‐fitted equivalent circuit yields *R*_S_, which, in principle, should equal the resistance of the corresponding “empty” junction (see Section 2.1.1), i.e., shorted but otherwise of identical geometry and configuration as the junctions used to measure the samples. However, in practice, this common‐sense identity is not always observed, specifically for junctions with the hanging EGaIn cone electrode, where the impedance‐derived *R*_S_ deviated by several orders of magnitude from the DC‐shorted (DC‐*R*_S_) junction one. The reason for this discrepancy could be limitations of the impedance technique applied to junctions with a semiliquid cone‐shaped top electrode as contact (see Section 2.1.2 for details).

**Scheme 2:**
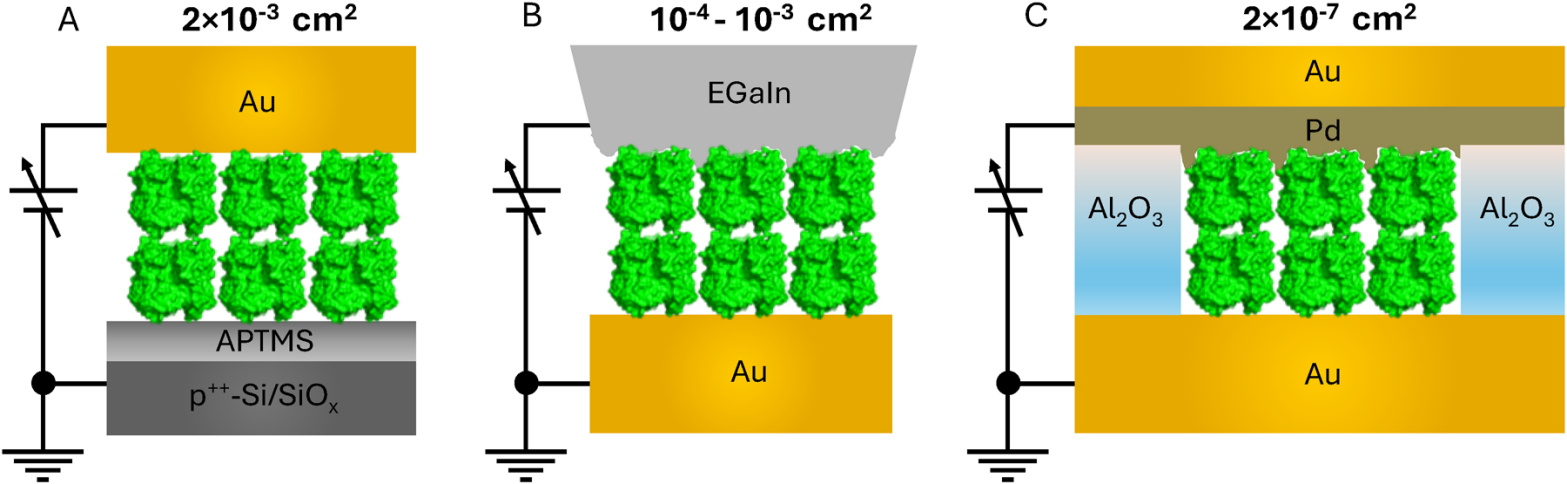
Three different device configurations of both human serum albumin (HSA) and bacteriorhodopsin (bR) junctions with a cartoon representation of protein layers A): p^++^‐Si(SiO*_x_*)/APTMS/protein/Au^pad^ (Si–Au), B): Au/linker/protein/EGaIn^cone^ (Au–EGaIn), and C): Au/linker/protein/(Pd–Au)^MpD^. The respective geometric junction areas are indicated above the scheme of each device. Here, HSA binds directly to the bottom Au‐electrode without a linker layer (via exposed cysteine residues), whereas bR is immobilized on Au via a cysteamine linker in both the Au–EGaIn and micropore device (MpD) configurations.

Here, an approach is presented to quantify the influence of $\hat{R_{C}}$ by comparing both DC and AC transport measurements of systematically varying protein multilayers and testing its application on three distinct protein junction configurations (see **Scheme** **2**):

p^++^‐Si(SiO*_x_*)/linker/protein/Au^pad^, Si–Au;

Au/linker/protein/EGaIn^cone^, Au–EGaIn; and

Au/linker/protein/(Pd/Au)^MpD^, micropore device (MpD).

The Si–Au and MpD junctions exhibit good reproducibility between AC and DC measured values (detailed methodology is given in Section S2, Supporting Information), while for the Au–EGaIn junction, here the DC measurements are exclusively used due to the inherent impedance limitations for the hanging‐cone‐EGaIn‐electrode‐based protein junction (see Section 2.1.2 in the main text and Section S2.2.2 in the Supporting Information).

To establish the validity of the $\hat{R_{C}}$ method and genuine variation in protein transport properties, two distinct protein systems were investigated. The first is HSA, a cofactor‐free globular protein, chosen as a model system due to its advantages in junction preparation (discussed in Section S3, Supporting Information). In parallel, we examined bR, a retinal chromophore‐embedded membrane protein. The bR system was recently reported to show unusual near‐activation‐less electron transport over 60 nm thick multilayer protein samples in a Si–Au configuration.^[^ ^2^ ^]^ Our key finding is that the MpD configuration effectively eliminates $\hat{R_{C}}$ for the protein junctions, which enables getting at intrinsic protein transport characteristics. In a general way, this suggests that $\hat{R_{C}}$ can be eliminated or at least minimized by selecting such MpD‐based metal/protein/metal junction. Additionally, several published studies have been reanalyzed, ranging from small molecules to peptides to test the validity of our approach. Our results show the dominant role of interface electrostatics, while direct or linker‐mediated protein–electrode contact, effective electrical contact, junction area, and electrode conductivity have a smaller influence on $\hat{R_{C}}$.

## 2 Results and Discussion

In the Supporting Information, a comprehensive protocol has been provided for the preparation of the HSA and bR protein layers (Sections S4 and S5, Supporting Information) and for device fabrication (see the Experimental Section), including their surface and electrical characterizations (Sections S6–S10, Supporting Information).

### 2.1 Insights into Protein Circuit Elements

The fundamental circuit model of the dry protein junction is depicted in Scheme 1. A simplified equivalent circuit for the protein junction^[^ ^2^ ^]^ was constructed based on impedance fitting,^[^ ^27^ ^]^ as elaborated in Sections S1 and S2.2 (Supporting Information). Furthermore, the reliability of our experimental impedance data was tested using the Kramers–Kronig test (Section S1.2 and Figure S1, Supporting Information).

#### 2.1.1 Concept of Empty Junction

In this study, the term “empty junction” refers to a configuration where no molecular or protein layer is present between the electrodes. Although such junctions may still include thin oxide layers from the electrodes (e.g., SiO_x_, GaO_x_), contributions of those are taken as part of the overall electrode resistance. We examined two types of protein‐depleted empty junctions to extract their resistance characteristics. The first is a real, experimentally obtained shorted junction, where resistance was measured directly using both DC (yielding DC‐*R*_S_) and *R*_S_ (AC) from impedance. These values primarily reflect contributions from the external wiring and the intrinsic properties of the electrode materials, as confirmed by impedance fitting. The second type is a virtual empty junction,^[^ ^25^ ^]^ which cannot be realized experimentally. It is obtained by extrapolating resistances of protein junctions of different lengths, to zero length, expressed as *R*_ZLR_. This extrapolated “zero‐length resistance” is independent of the protein layer's length but can, apart from electrode and wiring resistances, still include contributions from the electrode–protein interface (i.e., resistance that has its cause in the protein–electrode contact). The difference between the resistance of real and virtual empty junctions offers insight into the contact resistance specific to a given protein (or, in general, molecule or any other chemical species) junction type. This comparison forms a basic aspect of our analysis, its significance and implications are being elaborated in the subsequent sections.

#### 2.1.2 Insight into *R*_S_

**Figure 1:**
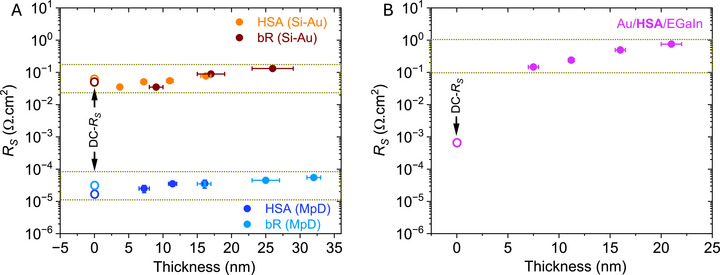
Impedance‐derived, averaged *R*_S_ (AC) values for different protein junctions (including both HSA and bR, with/without linker) are shown by color‐filled circular dots, while the DC‐*R*_S_ values for the bare devices (shorted junction) are represented by hollow circular‐color dots derived from *J*–*V* slope (DC‐measurement). A) Different protein junctions with Si–Au and MpD device configurations are indicated in figure legends with the proper color. B) For Au/HSA/EGaIn junctions.

In this study, *R*_S_ is a critical parameter for evaluating contact resistance and assuring the reliability of impedance measurements. We use *R*_S_ as “series resistance,” in line with standard conventions in impedance analysis.^[^ ^28^ ^]^ At high frequencies (≈1 MHz), the total junction resistance (*R*_T_) is effectively reduced to *R*_S_ because the AC current primarily flows through the capacitor, shorting *C*_P_. In the parallel *R*–*C* circuit, under high frequency condition, there is negligible contribution of capacitive reactants (*X*_C_) in Si–Au and MpD configurations (with EGaIn cone contact in our Au–EGaIn configuration, the contribution can be significant; see Section S2.2.2 in the Supporting Information) while *R*_P_​ presents a very high resistive path, blocking the current (see Scheme 1). From DC measurements of shorted junctions (without a protein layer), the inverse slope of the *J*–*V* curve near zero voltage (see Figure S2 in the Supporting Information) yields the DC‐*R*_S_ value (see Section S11 in the Supporting Information). Notably, DC‐*R*_S_ closely aligns with the impedance‐derived *R*_S_ value (see **Figure** **1**), confirming that *R*_S_ reflects the properties of the terminal leads and external wire connections, independent of protein–electrode contact (see Sections S1.1 and S1.3 in the Supporting Information). Based on our results, *R*_S_ is a property of a particular device configuration; it is independent of the nature of the junction material and the protein–electrode contact (details discussed in Sections S1.1 and S1.3, Supporting Information).

To account for variations in junction area, *R*_S_ is presented in an area‐normalized format, as it is primarily influenced by the dimensions of the junction.^[^ ^29^ ^]^ Here, all the resistance components are represented in an area‐normalized fashion based on the geometric area of the smallest contact of a junction. The extracted *R*_S_ values follow the trend MpD < Au–EGaIn < Si–Au (see **Table** **1**), a reasonable trend from the sum of the expected resistances of the two contacts. Given that one terminal electrode in each configuration is gold (Au), the observed differences in *R*_S_ are attributed to the resistivity of the other electrode (Au/Pd for MpD < EGaIn < p^++^‐Si). For HSA and bR junctions, DC and AC *R*_S_ values in the Si–Au and MpD configurations agree within a factor of 3 for a given protein or between proteins for a given contact configuration (Table 1, Figure 1).

**Table 1:** Averaged *R*_S_ values for various protein junctions, derived from impedance fitting, and the reciprocal slope of the *J*–*V* curves (see Section S11 in the Supporting Information) from DC measurements of electrically shorted junctions of the type indicated for each line.

Junctions	*R*_S_ [Ω cm^2^]	
	Impedance derived (fitting)	DC measurement (shorted junction)
Si/SiO*_x_*/APTMS/HSA/Au‐pad	5.0E−2 ± 2.0E−2	6.0E−2 ± 3.0E−2
Si/SiO*_x_*/APTMS/bR/Au‐pad	9.0E−2 ± 5.0E−2	3.0E−2 ± 2.0E−2
Au/HSA/EGaIn	4.0E−1 ± 3.0E−1	7.0E−4 ± 3.0E−4
Au/HSA/Pd–Au(MpD)	3.0E−5 ± 2.0E−5	1.0E−5 ± 2.0E−5
Au/cys/bR/Pd–Au(MpD)	2.0E−5 ± 3.0E−5	3.0E−5 ± 3.0E−5

By contrast, impedance‐derived *R*_S_ values for Au–EGaIn junctions deviate significantly (>10^3^) from DC‐*R*_S_ (Figure 1, Table 1). This suggests a fundamental origin for the difference between AC and DC measurements on junctions with hanging EGaIn‐cone top electrode. A likely origin is the problem to maintain a stable protein (/molecule) EGaIn cone interface with a fixed electrical contact area,^[^ ^30^ ^]^ as that area may fluctuate during the measurement due to limited mechanical stability of the EGaIn‐cone electrode (discussion in Sections S2.2.2 and S1.3.1 in the Supporting Information). Dynamic variations at the protein–EGaIn interface during frequency sweeps, coupled with insufficient adhesive forces between protein layer and the EGaIn cone, likely contribute to this phenomenon. Even for Au/EGaIn junctions without any molecules (“empty” junction), the significant difference was found between impedance and DC‐derived *R*_S_ values is consistent with some mechanical instability of the EGaIn cone and sample/other electrode surface during frequency sweeps, which interfere with impedance measurements. Likely, encapsulated or microchannel‐stabilized EGaIn contacts can offer significantly enhanced mechanical and electrical stability.^[^ ^12^, ^31^, ^32^, ^33^ ^]^ Given the instability issues associated with the cone‐shaped EGaIn contact, we focused exclusively on DC‐based measurements with that contact for our analyses. While DC values give the time‐ and space (contact areas)‐averaged results of a steady state that is established for the applied bias, reaching a steady state becomes problematic with AC modulation. Consequently, impedance‐derived *R*_S_ values for Au–EGaIn junctions become unreliable for comparative analysis (see more in Section S2.2.2 in the Supporting Information). Therefore, throughout this study, DC‐based measurements were exclusively used for these junctions.

#### 2.1.3 *R*_P_ and Interface Contributions

At low frequencies (<10 Hz), AC current traverses both resistive components in the equivalent circuit, while the capacitor (*C*_P_) behaves as an open circuit, effectively blocking AC current. Consequently, the total junction resistance is measured as *R*_T_ = *R*_S_ + *R*_P_ (a series combination) (see Scheme 1). *R*_P_ values were extracted from the AC measurements via modeling of the Nyquist plot, based on the proposed equivalent circuits (Scheme 1) for each protein junction (see Section S1 in the Supporting Information). In parallel, DC measurements provided an alternative estimate of *R*_T_ (from the average *J*–*V* response obtained from Figure S3, described in Section S11 in the Supporting Information), where the impedance‐derived *R*_P_ closely matched the DC‐derived *R*_T_ for both HSA and bR in Si–Au and MpD configurations (see Figure S4 in the Supporting Information). This convergence supports the reliability of our impedance results and indicates that contact effects can be reasonably understood also in terms of the length‐dependent *R*_T_. Thus, with a simple DC‐derived length‐dependent junction resistance model and extrapolation to zero junction length as an alternative method for the investigation of length‐dependent *R*_P_ (impedance derived), we arrive at the same conclusion as from impedance data about $\hat{R_{C}}$ effects in the junction characteristics (on *R*_P_) (see later part of this section). This agreement arises because *R*_T_ is predominantly governed by *R*_P_, with only a negligible contribution from *R*_S_ (i.e., *R*_P_ ≈ *R*_T_). However, as mentioned earlier (Section 2.1.2), due to experimental limitations in Au–EGaIn (EGaIn‐cone) junctions, the total resistance (equivalent to *R*_P_​) can be reliably determined only through DC measurements and used for further analysis.

**Figure 2:**
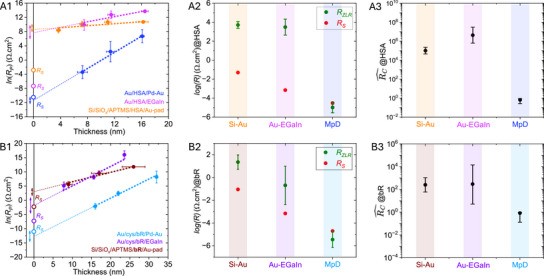
A1, B1) Plots of ln(*R*_P_) versus protein layer thickness, to estimate parameters for the three different types of protein junctions, each with (HSA or bR). The different devices are distinguished by their colors. The linear fits (dotted lines; *R* ^2^ = 0.99) yield the *Y*‐axis intercept, representing *R*_ZLR_. The colored double‐headed arrows indicate experimental errors (mean ± standard deviation), while the colored hollow circular dots at zero length correspond to *R*_S_ of respective devices. Both *X* and *Y* error bars represent the mean ± standard deviation over 20 protein junctions per data set. The error bars for *R*_S_ fall within the represented dots. A2, B2) The relative contributions of *R*_S_ and *R*_ZLR_ are illustrated; A3, B3) The estimated $\hat{R_{C}}$ values (cf. Equation (1))​ for a specific junction and protein type are provided.

Strikingly, for both HSA and bR junctions (Figure S4, Supporting Information), switching from Si–Au to MpD configurations led to a decrease of several orders of magnitude in *R*_P_, particularly for the thinner (8 and 16 nm) protein junctions (**Figure** **2**). This finding indicates that *R*_P_ is not solely dictated by the intrinsic properties of the protein layer. Instead, there are likely significant contact effects, arising primarily from the protein–electrode interface. Thus, *R*_P_ includes contributions from both the bulk protein and the protein–electrode contacts. Still, the $\hat{R_{C}}$ cannot be included as a resistance in series with *R*_P_, because in that case, the total resistance would be the sum of $\hat{R_{C}}$ and *R*_P_. Instead, $\hat{R_{C}}$ represents the contact limitation of the conduction across the protein‐based junctions, instead of a typical resistor component. We view $\hat{R_{C}}$ as a factor (not a sum), governed by the electrode and the electrode/protein interface; for example, the resistance posed by electron injection from/into the contact would be a multiplier. To mitigate interface effects, an extensive contact‐engineering study was conducted, measuring electron transport (ETp) across HSA and bR junctions in each of the three distinct device configurations.

The results of our impedance measurements on the different protein junctions consistently display clean semicircular Nyquist plots without Warburg behavior (Figure S5, Supporting Information), indicating the absence of ionic transport and confirming purely electronic conduction in our protein junctions under high vacuum (<10^−5^ mbar) conditions. The MpD configuration, with full metal encapsulation, effectively prevents moisture ingress and preserves the structural integrity of the protein layer. A rigorous drying protocol, dry N_2_ flow followed by overnight evacuation below 10^−7^ mbar prior to top electrode deposition, ensures removal of unbound water.^[^ ^14^ ^]^ While some buffer ions and tightly bound structural water remain, these are intrinsic to the protein and cannot migrate. As demonstrated in parallel work,^[^ ^14^ ^]^ proteins remain functionally intact after electrode deposition. Together, these observations confirm that residual solvent does not significantly influence the measured charge transport properties.

### 2.2 Length‐Dependence of *R*_P_ and Introduction of *R*_ZLR_ and $\hat{R_{C}}$

The resistance of protein‐based junctions (*R*_P_ or *R*_T_) depends exponentially on protein layer thickness (*d*), following a general trend irrespective of experimental device configurations (Figure 2). Extrapolating to *d* = 0 reveals a finite resistance at the *y*‐axis intercept, termed the *R*_ZLR_. Empirically, *R*_P_ can be expressed as

(2) $$R_{P}=R_{ZLR}\;.e^{\beta .d}$$

where *β* is a distance decay constant (see **Table** **2**). Given the junction lengths of ≈20 nm and above, well beyond the accepted quantum tunneling lengths for a mostly nonconjugated organic medium as the proteins, *β* cannot be described as the tunneling length decay parameter. This is so, even though currents are temperature independent down to ≈10 K,^[^ ^2^, ^14^ ^]^ as will be described in detail elsewhere. Here, the *β* is treated as a phenomenological length decay parameter, without connection to a specific transport mechanism; *R*_ZLR_ serves as the pre‐exponential factor. In this study, *R*_P_ values were derived from impedance fitting for protein‐based Si–Au and MpD junctions, while for Au–EGaIn configurations, DC‐derived total resistance (*R*_T_ ≈ *R*_P_, given *R*_P_ ≫ *R*_S_) was used. The estimated *R*_ZLR_ values for different protein types and configurations are presented in Table 2.

**Table 2:** Parameters, extracted for the different protein junctions, from ln(*R*_P_) versus thickness plots from Figure 2. A: HSA; B: bR. Here, the $\hat{R_{C}}$ values are unitless as they refer to the relative deviation of *R*_ZLR_​ from *R*_S​_ for a specific junction and protein type (cf. Equation (1)).

Table 2A			
Junctions	*R*_ZLR_ [Ω cm^2^]	$\hat{R_{C}}$	*β* [nm^−1^]
Au/HSA/Pd–Au	3.2E−6–3.4E−5	8.0E−01	1.10 ± 0.05
Au/HSA/EGaIn	4.4E+2–2.1E+4	4.4E+06	0.35 ± 0.10
Si/SiO*_x_*/APTMS/HSA/Au‐pad	2.3E+3–1.2E+4	1.0E+05	0.10 ± 0.05
**Table 2B**			
Junctions	*R*_ZLR_ [Ω cm^2^]	$\hat{R_{C}}$	*β* [nm^−1^]
Au/bR/Pd–Au	8.1E−7–1.5E−5	9.6E−01	0.70 ± 0.05
Au/bR/EGaIn	4.3E−3–1.0E+1	2.9E+02	0.70 ± 0.10
Si/SiO*_x_*/APTMS/bR/Au‐pad	5.4E+0–9.6E+1	2.5E+02	0.30 ± 0.05

**Figure 3:**
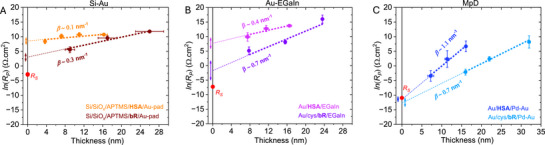
Comparing HSA and bR junctions in terms of the ln(*R*_P_) versus thickness plots in all three device configurations, as indicated in the figure legends with distinct color schemes; the slope measures respective *β* values (nm^−1^). The red dot is the averaged series resistance (the error bars fall within the represented dots); a double‐headed arrow indicates the estimated *R*_ZLR_ (with experimental errors; mean ± standard deviation) by linear fitting. Separate device configurations are A) Si–Au, B) Au–EGaIn, and C) MpD.

For HSA junctions, the average *R*_ZLR_ is approximately two orders of magnitude higher than for bR junctions in Si–Au configurations, and four orders of magnitude higher in Au–EGaIn setups (**Figure** **3**). However, MpD junctions exhibit significantly lower *R*_ZLR_ values, with minimal variation between bR and HSA. *R*_ZLR_ should predominantly reflect resistance contributions from leads and external circuitry, independent of the protein layer. Thus, it should ideally converge with *R*_S_. However, deviations are evident across different configurations: while MpD junctions show near‐convergence, Si–Au and Au–EGaIn setups exhibit substantial discrepancies. As noted above (see Introduction section), this difference is represented by the dimensionless form of $\hat{R_{C}}$ (see Equation (1)), i.e., the greater the difference **|** *R*_ZLR_ − *R*_S_ **|**, the higher is value of $\hat{R_{C}}$. It is reasonable to attribute the variations in $\hat{R_{C}}$ to chemical and electrical protein–electrode interactions, which will be affected by differences in protein surface charges. Notably, $\hat{R_{C}}$ arises from interfacial electrostatic interactions,^[^ ^21^, ^34^ ^]^ which can be due to the top or bottom interface, or both, is significant in Si–Au and Au–EGaIn junctions, where it constitutes a major part of *R*_P_. By contrast, in MpD configurations *R*_ZLR_ ≈ *R*_S_, i.e., protein/contact interactions have minimal impact (see Figures 2 and 3C).

### 2.3 Origin of $\hat{R_{C}}$ in Protein Junctions

The concept of “conductance” in molecular systems, including proteins, is often treated by analogy to bulk materials like copper or silicon. However, a fundamental question in ETp studies arises: do we truly measure the intrinsic properties of the protein, or does $\hat{R_{C}}$ play a dominant factor? Our results demonstrate that in MpD junctions, $\hat{R_{C}}$ is minimized for the two proteins studied here, by using high free electron density metals as contact materials and vacuum deposition. Evaporation of a metal such as Pd enables dense, stable small area contacts, formed by small‐sized particles with minimal chance of penetrating the protein film.^[^ ^14^ ^]^ Interfaces involving oxides (e.g., Si–Au‐pad, Au–EGaIn) significantly affect $\hat{R_{C}}$, likely due to electrostatic interaction between oxide and protein, with the surface charge density of the latter dictated by the pH of the solution used for self‐assembly. Such effects are mitigated in contacts with high free‐electron density metals. Additionally, the presence of a short (<1 nm) molecular linker in MpD setups has negligible influence on $\hat{R_{C}}$, underscoring the generality of this approach.

#### 2.3.1 Influence of $\hat{R_{C}}$ on ETp

Our experimental findings reveal that in ETp through both Si–Au and Au–EGaIn protein junctions, $\hat{R_{C}}$ is substantial. In Si–Au configurations, the relative deviation of *R*_ZLR_ » 10^5^ *R*_S_ for HSA and »10^2^ *R*_S_ for bR. In Au–EGaIn junctions *R*_ZLR_ » 10^6^ *R*_S_ for HSA, while for bR, *R*_ZLR_ remains as in Si–Au junctions, »10^2^ *R*_S_ (Figure 2 and Table 2). Within the proposed circuit model (Scheme 1), $\hat{R_{C}}$ can be positioned before or after *R*_P_, but impedance analysis cannot distinguish between the two separately. Consequently, in the presence of significant contact resistance, *R*_P_ is strongly affected, leading to $\hat{R_{C}}$‐dominated transport behavior.

While we cannot pinpoint the dominant origins of $\hat{R_{C}}$, interface‐specific factors are likely contributors. In Si–Au junctions, an ≈1 nm silicon oxide^[^ ^2^ ^]^ layer at the bottom interface increases resistance, while in Au–EGaIn junctions, an ≈1 nm Ga‐oxide^[^ ^12^, ^31^ ^]^ layer, whose resistivity is ≈10^8^ times^[^ ^35^, ^36^ ^]^ higher than that of EGaIn—similarly influences $\hat{R_{C}}$. Interestingly, the presence of an interleaved ≈0.5 nm amine‐terminated molecular protein–electrode linker for bR (see Sections S4.1 and S5.3 in the Supporting Information) does not significantly alter $\hat{R_{C}}$ in the MpD configuration, The role of a mechanically placed top electrode in modulating $\hat{R_{C}}$ remains uncertain and is discussed later in Section 2.4.1.

Notably, although both Si–Au and Au–EGaIn configurations are highly contact‐dominated, they still distinguish between different protein types (Figures 2 and 3). For HSA junctions, we deduce a $\hat{R_{C}}$ value that is 10^3^–10^4^ times higher than for bR ones, likely due to the known differences in protein surface electrostatics. Protein data bank analysis (see Section S12 in the Supporting Information) reveals that the exposed HSA surface is predominantly composed of polar and charged residues with minimal cavity areas, which is different from bR (Figure S6, Supporting Information). This high interfacial charge density (Figure S6, Supporting Information) may facilitate charge redistribution during transport, further amplifying $\hat{R_{C}}$. Similar to protein junctions, interfacial electrostatics in molecular junctions can play a critical role in determining electrical properties. For instance, switching from a simple alkyl thiol^[^ ^31^ ^]^ to a conjugated organic molecule^[^ ^12^ ^]^ led to a variation in *R*_ZLR_ or *R*_0_ in the literature^[^ ^12^, ^31^ ^]^ by more than three orders of magnitude, even within the same device configuration (microchannel‐based EGaIn and Au). These findings strongly indicate that the nature of the molecule–electrode interface affects *R*_ZLR_ or *R*_0_, and consequently, the contact resistance of the molecular junction.

### 2.4 $\hat{R_{C}}$ in Molecular Junctions

**Figure 4:**
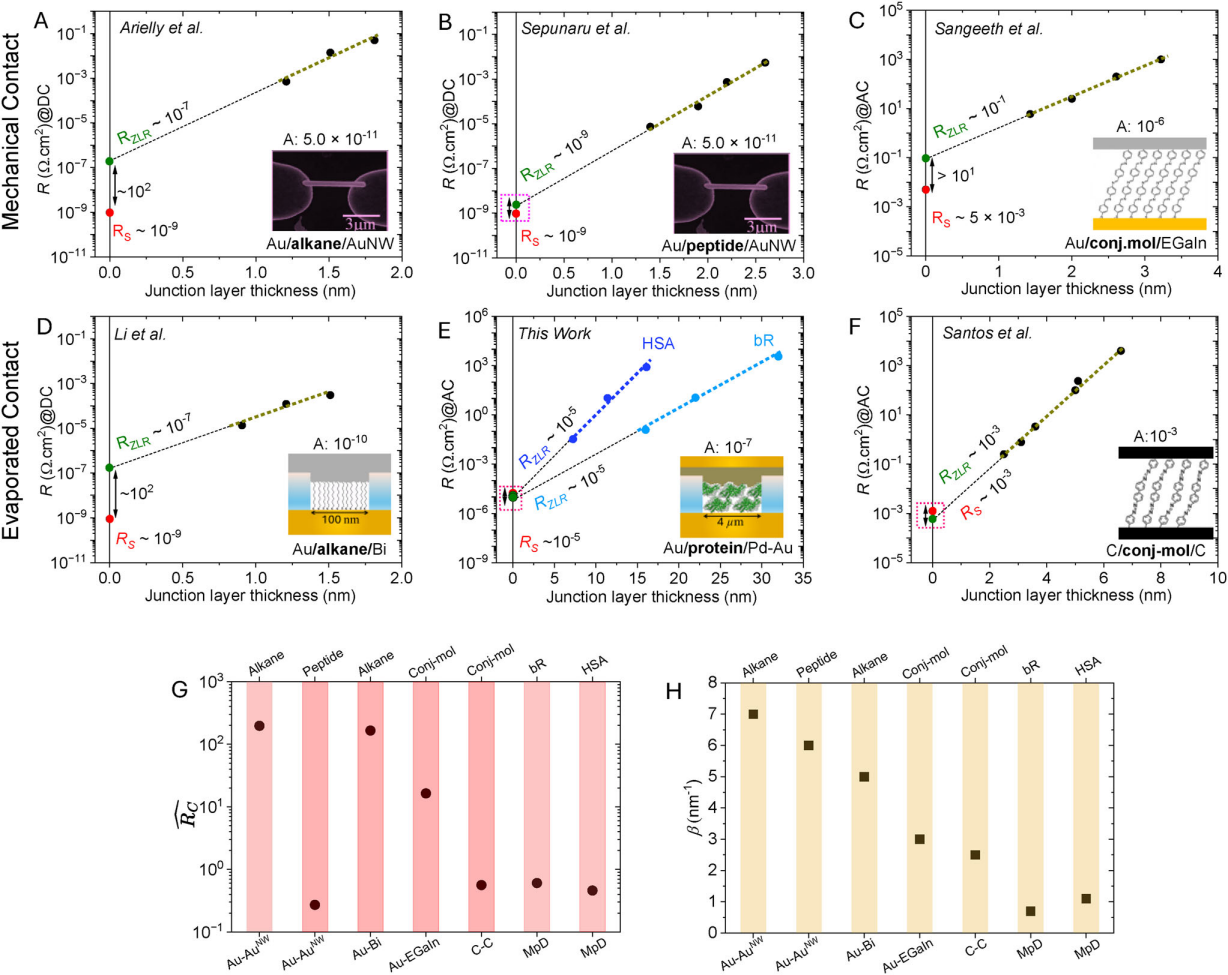
ln(*R*) versus *d* plots for A–D,F) various molecular junctions from the literature,^[^ ^12^, ^37^, ^38^, ^39^, ^40^ ^]^ as indicated in the figure legends, to extract the area‐normalized *R*_ZLR_ values; the geometric junction area [cm^2^] appears above the images/cartoons in each figure; E) the MpD results for both bR and HSA configurations. The red and green dots correspond to *R*_S_ and *R*_ZLR_ values in [Ω cm^2^], respectively, along with the averaged estimated values. Specific device configurations appear in the figure legends. Black arrows show |*R*_ZLR_ − *R*_S_|; a pink dotted box signifies the elimination of contact resistance. ROW‐I: mechanical top contact configurations; ROW‐II: evaporated top contact configurations. Panels (A),^[^ ^37^ ^]^ (B),^[^ ^38^ ^]^ and (D)^[^ ^40^ ^]^ show DC‐based data; panels (C),^[^ ^12^ ^]^ (E), and (F)^[^ ^39^ ^]^ show AC‐based data. Note: (A), (B), and (D) have the same ordinate; (C) and (F) have the same ordinate, while that for (E) is different as it spans 15 orders of magnitude, instead of 10 or 11. The estimated values of G)$\hat{\;R_{C}}\;$(cf. Equation (1)) and H) current decay constant (*β* nm^−1^) from the slope of ln*R* versus thickness for seven different molecular‐junction in published work including two protein junctions from this work, each featuring a distinct sandwiched molecular/protein layer (indicated at the top (G, H) along the *X*‐axis). The terminal junction electrodes are specified at the bottom of the figure.

To validate our approach for extracting $\hat{R_{C}}$ by disentangling it from *R*_P_, we reanalyzed length‐dependent resistance data from published literature.^[^ ^12^, ^37^, ^38^, ^39^, ^40^ ^]^ Studies on protein junctions remain sparse, and, instead, we examined various small organic molecular to oligopeptide junctions across different contact configurations, as summarized in **Figure** **4**. $\hat{R_{C}}$ values differ significantly between Au/alkyl–thiol/Au(nanowire)^[^ ^37^ ^]^ and Au/peptide/Au(nanowire),^[^ ^38^ ^]^ to be denoted both as “Au─Au^NW^,” junctions. The alkane‐based junctions exhibit an $\hat{R_{C}}$ of ≈100, whereas replacing alkane with a small peptide in the same Au─Au^NW^ configuration eliminates $\hat{R_{C}}$ (→ 0) (see Figure 4G). Similarly, conjugated organic molecules (conj‐mol) in Au/conj‐mol/EGaIn.^[^ ^12^ ^]^ “Au–EGaIn,” junctions show notable $\hat{R_{C}}$ (≈20), while carbon/conj‐mol/carbon^[^ ^39^ ^]^ (C─C) junctions display negligible $\hat{R_{C}}$.

#### 2.4.1 Influence of Junction Area on $\hat{R_{C}}$

As significant variations in $\hat{R_{C}}$ (spanning several orders of magnitude) arise across experimental configurations (Table 2), one can ask how far the differences in junction areas are responsible for these variations (up to 10^4^; Scheme 2), along with the nature of the top electrode contact. Note that 2 types (Si–Au, Au–EGaIn) are mechanically placed in the ambient, while the other is vacuum‐evaporated (MpD) contacts. As is known in the field of electrical (metal–metal) contacts, any of these differences can affect the electrical quality of the contacts.^[^ ^9^, ^41^ ^]^ Previous research established that molecular transport properties depend on the ratio of electrically active to geometric contact areas, which increases with decreasing geometrical area, approaching unity for STM studies.^[^ ^18^ ^]^ Mukhopadhyay et al.^[^ ^15^ ^]^ further reported significant interlaboratory variations in junction current magnitudes (but not in the currents’ temperature‐dependence), highlighting effective electrical contact as a critical factor. To assess the area effect on $\hat{R_{C}}$, a comparative analysis was conducted on published data.

Despite an enormous (>10^8^) junction area difference between the junctions reported and varying top contact types (mechanical vs evaporated), Sepunaru et al.^[^ ^38^ ^]^ and Santos et al.^[^ ^39^ ^]^ showed negligible $\hat{R_{C}}$. This suggests that $\hat{R_{C}}$ is not dictated by the effective electrical contact area.^[^ ^33^ ^]^ Notably, in Au─Au^NW^ configurations, replacing peptides^[^ ^38^ ^]^ with alkyl thiols^[^ ^37^ ^]^ introduces contact resistance, indicating that the electrode–molecule interface dominates over the contact area in determining $\hat{R_{C}}$. Junctions with similar alkyl thiol molecules and comparable top contact areas yield nearly identical $\hat{R_{C}}$ (≈100), regardless of whether the top electrode is evaporated Bi (Au─Bi; *A*_geo_ ≈10^−10^ cm^2^, Li and Selzer)^[^ ^40^ ^]^ or electrophoretically deposited Au (nanowire with *A*_geo_ ≈10^−11^ cm^2^, Arielly et al.).^[^ ^37^ ^]^ Conversely, carbon/conj‐mol/carbon^[^ ^39^ ^]^ junctions with a substantially larger area (1.3 × 10^−3^ cm^2^) exhibit negligible $\hat{R_{C}}$, while Au/conj‐mol/EGaIn^[^ ^12^ ^]^ junctions with a 10^3^‐fold smaller area (10^−6^ cm^2^) show a small but non‐negligible $\hat{R_{C}}$ (Figure 4, Sangeeth et al.).^[^ ^12^ ^]^ Therefore, the effective electrical contact area and its variations do not dominate $\hat{R_{C}}$, whereas the interfacial characteristics do.

#### 2.4.2 Electrode Type Effects on $\hat{R_{C}}$

Interestingly, relatively high‐resistance terminal electrodes (*R*_S_ ≈10^−3^ Ω cm^2^) in carbon/conj‐mol/carbon junctions do not influence $\hat{R_{C}}$, whereas low‐resistance terminal contacts (*R*_S_ ≈10^−10^ Ω cm^2^) in Au/alkyl–thiol/Au(nanowire) junctions induce significant $\hat{R_{C}}$. This underscores the dominant role of the molecule–electrode interface. In carbon/conj‐mol/carbon junctions, the absence of an interface transport barrier due to possible direct C─C interactions likely minimizes $\hat{R_{C}}$. By contrast, in Au/alkyl–thiol/Au(nanowire) junctions, interface dipoles are likely present between the terminal methyl group and the Au (possibly due to the spillover electron density pillow effect),^[^ ^42^, ^43^ ^]^ contributing to contact resistance for the uncharged alkyl chains. However, charged molecules such as proteins and peptides can neutralize or reduce metal–molecule interface dipoles and electrostatic effects, thereby decreasing or eliminating $\hat{R_{C}}$. Thus, the intrinsic resistance of the electrode material, and even that of the molecular core, typically does not dominate the contact resistance. Instead, key contributing factors are the presence of interfacial oxide layers, the true (electrically active) contact area, the nature of the contact at surface asperities, and, in cases where surface charge or dipole densities exceed ≈10^11^ cm^−2^,^[^ ^44^ ^]^ the electrostatic potential landscape shaped by the net surface charge distribution.

### 2.5 Intrinsic *β* Values of Protein Junctions

Experimentally, *β* was estimated from the slope of the ln(*R*_P_) versus *d* plots in Figure 3 for different junction configurations. Our results do not only show a way to eliminate contact resistance in protein junctions but also provide an accurate determination of *β* by taking into account $\hat{R_{C}}$. With HSA, *β* is an order of magnitude higher in the MpD configuration than in the Si–Au one, while *β* values for HSA Au–EGaIn junctions are in between these. For bR junctions, *β* values appear less affected by contact configuration, with those in the MpD or EGaIn configurations roughly twice that in the Si–Au junctions (Table 2). The *β* value extracted for Au/bR/EGaIn junctions, which still include moderate contact resistance, closely matches that of Au/bR/Pd MpD junctions. This consistency highlights an important transport characteristic of protein‐based junctions: the length decay parameter associated with transport length (*β*) is generally insensitive to moderate contact resistance. Only in cases where interfacial contact resistance is particularly high does it significantly impact the *β* value. Most likely, interface electrostatics control the charge injection of carriers into/extraction out of protein junctions. The differences in *β* values reflect different interface charges.

Contact engineering plays a critical role in modulating charge transport through protein films. In HSA junctions within the Si–Au configuration, higher contact resistance weakens transport length‐dependence, resulting in lower *β* values, which inaccurately represent the protein's transport properties. Conversely, configurations with minimal contact resistance yield *β* values that more accurately reflect intrinsic protein transport characteristics. In the junctions dominated by contact resistance (Si–Au and Au–EGaIn), *β* values are initially higher for thinner films (see Table S1 and Figure S7 in the Supporting Information) but decrease with increasing thickness.^[^ ^2^ ^]^ By contrast, junctions with negligible contact resistance (e.g., MpD) exhibit consistent *β* values across varying film widths. Previously,^[^ ^2^ ^]^ we reported an unusually low *β* value (<0.1 nm^−1^) for a bR protein junction, and its variation with protein layer thickness suggests that those earlier observations were likely dominated by strong contact effects, rather than reflecting the intrinsic transport properties of the protein. By contrast, the MpD configuration employed in this study allows for a more accurate assessment of the proteins’ intrinsic electronic transport characteristics. Given the relatively long distances of transport involved, the *β* values reported here should be interpreted as indicators of the relative efficiency of long‐range transport within specific protein environments, rather than as direct evidence of a particular transport mechanism.

The higher *β* value observed for HSA (*β* ≈ 1.1 nm^−1^) than for bR (*β* ≈ 0.7 nm^−1^) MpD junctions suggests relatively more efficient electron transport through bR than HSA layers. In the Si–Au configuration, this is the opposite, which we ascribe to contact resistance effects. Compared to small organic molecules and oligopeptides (*β* ≈ 3–7 nm^−1^), proteins exhibit lower *β* values (≈1 nm^−1^ or less) even if, as is the case here, contact resistance is minimized (Figures 4G, 4H), reflecting their efficiency as electron transport media. Other than for proteins, there are only a few reports of molecular junctions, and most are only 1 to a few nanometers thick (thin compared to the protein films used here) with very low *β* values and unusual transport efficiency.^[^ ^11^, ^45^, ^46^ ^]^

The one exception concerns work on carbon/conjugated organic oligomer film/carbon junctions, which exhibit length and temperature dependences,^[^ ^47^ ^]^ similar to what we find here. Remarkably, those dependences persist particularly in photocurrent studies of encapsulated junctions that minimize contact resistance,^[^ ^39^ ^]^ yielding very low *β* values.^[^ ^48^ ^]^ It is noted, though, that the systems are quite different, because the organic oligomers have a fully π‐conjugated backbone that enables continuous electronic charge transport, whereas proteins contain only a few, spatially well‐separated, aromatic residues, and the peptide backbone lacks conjugation.^[^ ^49^ ^]^ Furthermore, in the photocurrent oligomer studies, excited states will be involved in the transport, while in our study, only ground‐state conduction is possible (dark conditions). Thus, now that is known that the remarkable length‐ and temperature‐dependences persist also if contact resistance can be neglected, thus it is postulated that the transport mechanism will be fundamentally different for proteins, materials with wide HOMO–LUMO gaps of >2.5 eV, which drives home the need to discover the mechanism(s) that make such efficiency possible.

## 3 Conclusions

This study highlights the critical role of contact/interface engineering in correctly assessing intrinsic electron transport through proteins junctions of ultrathin protein films. By systematically eliminating contact resistance as a resistance‐limiting factor in two‐probe configurations, it is demonstrated that interfacial electrostatics, interface dipoles, and insulating interfacial layers, rather than effective electrical contact area, primarily govern $\hat{R_{C}}$. Our findings reveal that conventional contact‐dominated configurations (e.g., Si–Au and Au–EGaIn ones) can obscure intrinsic transport properties, likely due to interfacial oxides. The micropore device (MpD) configuration allows to effectively isolate and preserve the inherent charge transport characteristics of proteins, while having much better reproducibility than the Au‐Au nanowire^[^ ^50^ ^]^ contact approach. By contrast, the presence/absence of a linker or related protein–electrode bindings in an MpD configuration, does not need to lead to contact resistance. The consistent distance‐dependent current decay observed in $\hat{R_{C}}$‐free protein junctions proves that proteins can function as robust^[^ ^14^ ^]^ charge transport media, comparable to or even surpassing ultra‐thin (organic) molecular junctions. Our approach offers a blueprint for designing molecular and bioelectronic interfaces with control over charge transport. Future research should integrate this $\hat{R_{C}}$‐free methodology with emerging bioelectronics to enable next‐generation protein‐based transistors, energy harvesters, and biomolecular computing. As such, this work paves the way for scalable, tunable protein‐based bioelectronics by linking fundamental charge transport studies to practical applications.

## 4 Experimental Section

### Protein Layer Preparation

In this study, HSA (Sigma) films were systematically prepared, highlighting their advantages in detail (see Sections S4 and S3 in the Supporting Information). Similarly, bR layers were fabricated following a well‐established protocol,^[^ ^2^ ^]^ with additional procedural details provided in Section S5 (Supporting Information).

### Protein‐Based Device Fabrication

Three distinct vertical protein‐based device architectures were fabricated for transport measurements, as illustrated in Scheme 2. The fabrication process for each configuration was briefly summarized as follows.

### Protein‐Based Device Fabrication—Si–Au Configuration

Ultrathin gold pads (≈2 × 10^−3^ cm^2^) were manually placed onto protein layers deposited on linker‐functionalized conductive silicon substrates, following previously established protocols.^[^ ^2^, ^51^ ^]^

### Protein‐Based Device Fabrication—Au–EGaIn Configuration

A freshly prepared EGaIn cone was precisely positioned onto the protein layer using a micromanipulator, forming a large‐area (≈10^−4^–10^−3^ cm^2^) top electrode at the protein–EGaIn interface. The setup is depicted in Figure S8, with additional details in Section S6.3 (Supporting Information).

### Protein‐Based Device Fabrication—MpD

A specialized protocol was developed for fabricating metal/protein/metal MpD,^[^ ^14^ ^]^ designed to produce nonshorted, transport‐active protein junctions. Recently, it is demonstrated that in the MpD configuration, the underlying protein layer retained its functional activity despite the presence of the evaporated top electrode.^[^ ^14^ ^]^ A photolithographically defined bottom Au electrode (≈ 2 × 10^−7^ cm^2^) was coated with protein layers, followed by successive E‐beam evaporation of Pd and Au to form the top electrode. Further details are provided in Section S6.2 (Supporting Information).

### Electrical Characterization

Electrical transport characteristics were investigated using both DC measurements and AC‐controlled impedance spectroscopy in a two‐probe configuration. A comprehensive description of the measurement protocols is available in Section S2 (Supporting Information).

## Conflict of Interest

The authors declare no conflict of interest.

## Author Contributions

S.B. and D.C. conceptualized and designed the experiments in collaboration with D.E. and M.S. Experimental validation was carried out by S.B., A.V., D.E., M.S., and D.C. S.B. was responsible for the design, fabrication, optimization, and characterization of HSA‐based devices and contributed to bR‐based device development. S.B. conducted all electrical measurements (DC and impedance) on Si–Au and MpD junctions and performed data analysis for all protein‐based devices. Additionally, S.B. developed the complete setup for EGaIn‐based top electrode measurements and carried out HSA‐based Au–EGaIn junction measurements. S.D. extracted and purified bR, prepared bR thin films, and conducted DC electrical measurements on bR‐based Au–EGaIn junctions. Paper writing was contributed by S.B., A.V., I.P., M.S., and D.C., with all authors actively participating in discussions and revisions.

## Supporting information



Supporting Information

## Data Availability

The data that support the findings of this study are available from the corresponding author upon reasonable request.
