# Supplementary material for: Without Contact Resistance, Proteins in Thin‐Film Solid‐State Junctions Can Be Efficient Electronic Conducting Materials
Source: Adv Mater. 2025 Sep 19;38(2):e07654. doi: 10.1002/adma.202507654 (PMC12783954; doi:10.1002/adma.202507654)
Supplement: Supplementary file 1 — Supporting Information [file ADMA-38-e07654-s001.docx]

Supporting Information

**Without Contact Resistance, Proteins in Thin-Film Solid-State Junctions can be Efficient Electronic Conducting Materials**

*Sudipta Bera**, *Ayelet Vilan, Sourav Das, Israel Pecht, David Ehre, Mordechai Sheves**, *and David Cahen**

S. Bera, S. Das, D. Ehre, M. Sheves, and D. Cahen

Department of Molecular Chemistry and Materials Science

Weizmann Institute of Science

Rehovot 7610001, Israel

Email: [sudipta.bera@weizmann.ac.il](mailto:sudipta.bera@weizmann.ac.il), [mudi.sheves@weizmann.ac.il](mailto:mudi.sheves@weizmann.ac.il), [david.cahen@weizmann.ac.il](mailto:david.cahen@weizmann.ac.il)

A. Vilan

Department of Chemical Research Support

Weizmann Institute of Science

Rehovot 7610001, Israel

I. Pecht

Department of Immunology and Regenerative Biology

Weizmann Institute of Science

Rehovot 7610001, Israel

**Supporting Information Index**

S1. Impedance-derived equivalent circuits for protein devices ………………………….…..(S3)

S1.1. Insight into *R_S_*……………………………………………………….…...(S3-S4)

S1.2. Kramers-Kronig test on Impedance data………………………………....…(S4)

S1.3. Verification of equivalent circuit for “empty” junctions…………….…..(S4-S5)

S1.3.1. Protein-free Au-EGaIn junction*…*………………………….….…..(S5)

S2. Electrical measurements ………………………………………………………….……...(S6)

S2.1 Direct current (DC) measurements …………………………………………....(S6)

S2.2. Impedance measurements ……………………………………………………..(S6)

S2.2.1. Limited impedance response acquisition for the EGaIn junctions(S6-S7)

S2.2.2. Cause behind the large deviation………………………………........(S7)

S3. Structural advantages of HSA …………………………………………………………....(S8)

S4. HSA thin film preparation ……………………………………………………………......(S8)

S4.1. HSA-SAM preparation ………………………………………………....….(S8-S9)

S4.2. Preparation of HSA multilayers ………………………………………….…....(S9)

S5. bR thin film preparation …………………………………………………………......(S9-S10)

S5.1. Substrate Cleaning ………………………………………………………........(S10)

S5.2. Deposition of linker layer ……………………………………………….........(S10)

S5.3. bR bilayer(s) preparation …………………………………………………......(S10)

S6. Procedure for the top electrode deposition ………………………………………..…….(S10)

S6.1. Si-Au Junctions …………………………………………………………….....(S10)

S6.2. MpD devices and probe connections ……………………………..….......(S10-S11)

S6.3. EGaIn-cone-based top electrode landing and measurement setup …………....(S11)

S7. Quality and thickness of HSA/bR films …………………………………………….(S11-S12)

S8. Quality assessment of protein-based devices via impedance analysis ……………......…(S12)

S9. Inverse capacitance-HSA thickness relation ………………………………...…………..(S13)

S10. Monitoring protein layer thickness ………………………………………………..…...(S13)

S10.1. Ellipsometry ………………………………………………………….….…..(S14)

S10.2. AFM Scratching …………………………………………………………..…(S14)

S11. Junction resistance from *J-V* slope ………………………………………………….....(S14)

S12. Protein structural analysis ………………………………………………………..…....(S14)

Figures and Tables…………………………………………………….…….….(S15-S30)

**S1. Impedance-Derived Equivalent Circuits for Protein Devices**

A common way for presenting impedance response is the Nyquist plot, which displays the imaginary component (−*Z*_im_) versus the real component (*Z*_re_) of impedance across a range of AC frequencies. For all three device configurations, the Nyquist plots of the protein junctions exhibit near-perfect semicircles, indicative of a single dielectric relaxation in the protein layers, as demonstrated for both HSA and bR junctions (Figure S5). A single peak in the *–Z*_Im_ vs. frequency plot agrees with a single dielectric relaxation process (see Figure S9) occurs across all experimental junctions. Similar semicircular responses were observed in recent studies of bR junctions in Si-Au configurations.^[1]^ These impedance characteristics can be modeled using a simple equivalent circuit^[2,3]^: a parallel resistor-capacitor (*R*–*C*) element in series with a series resistance, *R*_S_ (see Scheme 1 in the main text). Here, *R*_P_ represents the "protein resistance", influenced by interfacial contact, protein type, and layer thickness. *C*_P_ denotes the "protein junction capacitance", which depends on the protein’s dielectric constant, electrode separation, and junction area. *R*_S_ accounts for the "series resistance" of the entire setup, including contributions from terminal electrodes but excluding effects from the protein or contact interface.

**S1.1. An Overview of *R*_S_**

An extensive control experiment using a Si–Au pad configuration with well-defined geometric areas of the junctions and several different materials between the electrodes was designed and performed. The materials used included layers of both proteinaceous (e.g., bR, HSA, etc.) and non-proteinaceous ones. The latter were poly-ionic polymer multilayers or ALD-grown ~2 nm SiO_x_ (instead of the standard chemically re-grown ~1 nm, used for all other Si substrates in this work). For all of these, we observe a narrow *R*_S_ distribution (^max^_/min_ < 2.5; cf. Table S2, and ^max^_/min_ < 4 for multilayers of a given protein with different junction thickness; cf. Figure 1 in the main text). Such a small variation can be accounted for by varying effective electrical contact areas due to differences in the roughness of the surface between protein films with different numbers of layers. These results support that *R*_S_ is largely independent of the sample-contact interfacial properties and instead reflects primarily reproducible system resistance. Moreover, the area-normalized DC resistance of the Si-Au-pad junction (the shorted, “empty” device) closely overlaps the *R*_S_ values from fitting the impedance spectroscopy data. This strong correlation across different protein/other materials’ junctions in Si-Au configuration, with different sets of contacts, supports our assertion that *R*_S_ is not significantly influenced by junction contact resistance. A similar trend was also observed for MpD junctions, as shown in the main text (Figure 1). Consistency is also observed in a separate set of crosswire devices of Au/protein/C junctions: impedance results from two distinct protein systems (bR and PS-I) give *R*_S_ values with ^max^_/min_ < 1.5 (data not shown; unpublished).

Therefore, by analyzing several distinct device configurations, *R*_S_ (AC) and DC-*R*_S_, we do not find experimental evidence to support a significant contribution to *R*_S_ of a component that is specific to the sample-contact combination, the contact resistance; rather, *R*_S_ is governed by the external wiring contributions, and by the bulk electrode materials' properties.

**S1.2. Kramers-Kronig (KK) Test on Impedance Data**

To ensure the reliability of our experimental impedance data for various protein junctions, particularly in Si-Au and MpD configurations, we conducted the KK test.^[4,5]^ All stable junctions gave semi-circular Nyquist plots and fit well with the proposed equivalent circuit (see Figure S5). Among the Au-EGaIn cone junctions, only the Au/HSA(bilayer; ~7 nm)/EGaIn junction consistently produced impedance signals with a satisfactory fit. The KK test was applied across the full dielectric spectrum to assess data validity and detect potential experimental artifacts such as noise, drift, or non-equilibrium conditions. This test numerically transforms the imaginary part of the impedance spectrum to calculate the theoretical real part (and *vice versa*), which is then compared with the measured values. The deviation between calculated and measured data, expressed as ‘%*Z*_Error_’ for both *Z*_Re_ and *Z*_Im_ components, was evaluated using *ZView 4* software.

The results showed strong linearity, confirming assumptions of thermodynamic equilibrium and linear system behavior. Most *Z*-errors remained within 10%, indicating high data fidelity (see Figure S1). For completeness, corresponding Nyquist plots with fitted curves are also provided in the SI Figure S1.

**S1.3. Verification of Proposed Equivalent Circuit for “Empty” Junctions**

To rule out any potential doubt, we analyzed both AC and DC current responses of the “empty” (shorted) devices using back-to-back measurements. Ideally, a shorted device, without a protein or molecular layer in the junction, should behave as a simple resistor. Accordingly, the expected Nyquist plot should be reduced to a single point, and the impedance phase angle should remain near zero across all frequencies. This is the behavior that we find for the MpD configuration and is very close to that of the Si-Au system. However, a notable deviation was found for the EGaIn cone electrode (Au-EGaIn) configuration.

For the shorted MpD junction, direct metal-to-metal contact exists without any dielectric barrier (see Figure S10). This is clearly reflected in our experimental data: a single-point Nyquist representation, absence of dielectric features in the -*Z*_Im_ vs. frequency plot, and an impedance phase that remains nearly zero across all frequencies. Furthermore, AC and DC resistance (*R*_S_) values show strong agreement, matching closely. This consistency confirms that *R*_S_ reflects the external circuitry and properties of the leads; it is not affected by interfacial resistance. For the Si-Au configuration, a similarly simple response was observed with minor deviations. These are attributed to a thin (~1 nm) native SiO_x_ layer, which is not fully continuous; it appears leaky, as is evident from a modest increase in high-frequency impedance phase, peaking around 6°, indicating partial dielectric behavior. Still, AC *R*_S_ and DC-*R*_S_ values overlap closely, reinforcing the reliability of *R*_S_ as a meaningful resistance measure across these different protein junctions.

*S1.3.1. Protein-free Au-EGaIn Junction*

To further investigate the intrinsic impedance behavior of the EGaIn cone contact configuration, we performed a series of controlled experiments using bare Au substrates, specifically focusing on junctions without any protein or linker layer. In these experiments, both impedance (AC) and I–V (DC) measurements were conducted sequentially on the same junction to ensure consistency. Notably, we observe a significant difference between the AC and DC resistance (*R*_S_) values, with the impedance-derived *R*_S_ being approximately 20× higher than the DC *R*_S_. The underlying reasons for this deviation are discussed in detail in Section S2.2.2. Our setup employs a EGaIn cone contact (by far the most widely used and popular EGaIn contact configuration in the field), and possibly, the thickness and roughness of its oxide differ from those for other EGaIn contact approaches.

Applying Ockam’s razor, we attribute the observed dielectric behavior in nominally "bare" Au/EGaIn junctions to the presence of at least a partial GaO_X_ interfacial layer. This interpretation is supported by several key experimental observations (see Figure S10).

- Nyquist plots show a single, well-defined semicircle, indicative of a single dielectric relaxation process.
- −Z_Im_ vs. frequency plot exhibits a clear peak, further consistent with the presence of a dielectric GaO_X_ skin between the Au bottom electrode and bulk
- Impedance phase angle (*θ*_max_) exceeds 60°, which rules out a purely resistive interface and suggests the presence of a capacitive component. However, since *θ*_max_ remains below 90°, it implies leaky capacitive behavior, again pointing to GaO_X_.

These features collectively are not consistent with purely metallic contact. Although the junctions display Ohmic-like DC behavior, the impedance analysis reveals clear dielectric characteristics attributable to the native GaO_X_ interlayer. This control experiment confirms that even in the absence of proteins, the interleaved GaO_X_ layer significantly influences the junction’s electrical properties. Importantly, this dielectric layer serves as a physical barrier, preventing alloy formation between bulk EGaIn and the Au electrode within the experimental timeframe and junction configuration used (i.e., *µ*m-scale area with EGaIn cone electrode). If alloying had occurred, the impedance response would reduce to a simple resistor-like behavior, as seen in shorted MpD junctions (Figure S10). Therefore, the fact that the “empty” Au-EGaIn junctions still exhibit dielectric features also in the absence of proteins, leads to the conclusion that GaO_X_ plays a critical and persistent role in modulating the interface.

**S2. Electrical Measurements**

In this work, we utilized both DC and impedance (AC) measurements to characterize various experimental protein junctions in a two-probe configuration. Si-Au and MpD-based protein devices were measured in a probe station (*LakeShore TTPX*) under a vacuum range of 10^-3^ to 10^-5^ mbar at room temperature (293 ± 2 K). Due to practical constraints, the Au-EGaIn junctions were measured under ambient conditions (< 40 % RH), as detailed in SI Sections S2.2.1 and S6.3.

**S2.1. Direct Current (DC) Measurements**

DC measurements were carried out by varying bias voltages using a sub-fA source meter (*Keithley 6430*) with an applied bias voltage ranging from ±0.1 V (1 mV step value) to ±1.0 V (10 mV step value) to the top electrode, while the bottom electrode was grounded. We executed the voltage sweep in a proper voltage loop starting from a zero voltage, moving to positive voltage maximum, then to the negative bias end through zero bias, and finally back to 0 V, using dedicated LabView-based programmable software with a scan rate 2 mV/S.

**S2.2. Impedance Measurements**

Impedance spectroscopy (IS) measurements of the protein junctions were carried out at room temperature using a *Zurich Instruments MFIA* impedance analyzer. An AC bias with an amplitude ranging from 10 to 50 mV was applied with 0 V direct bias, and the frequency was swept from 1 Hz to 1 MHz at a rate of 15-20 data points per decade. The impedance data were analyzed in the form of Nyquist plots and phase vs. frequency (a part of the Bode plot). Equivalent circuit elements were constructed from experimental data fitting using *ZView 4* software (*Scribner Associates*). Our impedance data fit well to equivalent circuits, yielding high-quality fits with χ^2^ ≈ 10^-3^.

S2.2.1. *Limited Impedance Response Acquisition for the EGaIn Junctions*

The impedance signal was noisy for the thicker protein layers (and there has not yet been any report of IS experiments with EGaIn contact with protein layer, thicker than 10 nm), and only the bilayer junction of HSA yielded reasonable signals (see Figure S5C) under the 100 mV applied AC bias amplitude to improve the signal-to-noise ratio. For the EGaIn junction, stable impedance data could be recorded only at a high frequency of ~1 MHz, which approximately leads to the *R*_S_ of the junctions, and we also extracted the junction *C*_P_ values directly from the value of parallel capacitors in the equivalent circuit module of MFIA connected LabOne software setup for different HSA junctions. We haven’t tried this on bR junctions. The large deviation between *R*_S_ and DC-*R*_S_, we restricted our measurements to EGaIn junctions for both proteins, where we mainly focused on DC measurements.

*S2.2.2. Cause Behind the Large Deviation*

In general, the series resistance can be obtained from the high-frequency limit (here 1 MHz) of a fit to the impedance data. The real component of a parallel resistor-capacitor ($R$*,* $C$) couple, that is connected in series to a second resistor (*R*_S_), includes an effect of the capacitor (see circuit in Scheme 1 in the main text):

$Real\left( Z \right)=Z_{Re}=R_{S}+\frac{R_{P}}{1+\omega^{2}{R_{P}}^{2}{C_{P}}^{2}}$ (S1)

Here, the second term on the right-hand side is the real part of the capacitive reactance (*X_C_*)

$$X_{C}(Real)=\frac{R_{P}}{1+\omega^{2}{R_{P}}^{2}{C_{P}}^{2}}$$

$Img\left( Z \right)=Z_{Im}=-\frac{\omega{R_{P}}^{2}C_{P}}{1+\omega^{2}{R_{P}}^{2}{C_{P}}^{2}}$ (S2)

From Equation (S1), we get $R_{S}$, as $\omega\to\infty$:

$R_{S}\cong\lim_{\omega\to\infty} Z_{Re}$ (S3)

However, deriving $R_{S}$ from equation (S3), will be inaccurate if even at the highest measured frequency, $\omega_{\max}$ (2$\pi$ MHz in our case), the condition (*𝜔*$R_{P}C_{P})^{2}\gg\left( R_{P}/R_{S}-1 \right)$, is not met, i.e., the case of our EGaIn-cone contacts. The real part of the capacitive reactance is approximately one order of magnitude greater than the series resistance (*R*_S_) for the EGaIn cone junction. In both Si-Au and MpD, the high-frequency approximation reduces the net junction impedance to *R*_S_; for Si-Au, *X*_C_ is about 100× lower, and for MpD, nearly 10^4^ × lower than *R*_S_, which makes AC-*R*_S_ = DC-*R*_S_ (Figure 1 in the main text). These differences in capacitive reactance alone cannot account for the observed deviations between AC and DC measurements. An additional likely contributing factor is a possible time-dependent variation in actual electrical contact area,^[6]^ caused by the mechanical instability of the EGaIn cone electrode during frequency sweeps, which will affect impedance measurements and could lead to the experimental results; the exact cause remains unclear and is beyond the scope of the present study.

During DC measurements, capacitive reactants were absent, and the mechanical instability based on electrical contact area fluctuations did not influence prominently, likely because the actual junction area averages out (over 2-3 min) in the long-term DC measurements. Therefore, we relied intentionally on DC-based measurement results for estimating the contact resistance ($\hat{R_{C}}$), as the impedance data proved unreliable in our measurement setup.

**S3. Structural Advantages of HSA**

Human serum albumin (HSA) possesses several features that make it highly suitable for transport studies. As a polypeptide-only protein, it readily forms mono- and multilayer junctions, aided by its numerous surface-exposed functional groups, such as cysteine, aspartic acid, glutamic acid, and lysine, which facilitate diverse substrate-protein linkages and inter-protein coupling (Figure S11). Additionally, HSA exhibits a unique binding affinity for various molecules, including ions, retinoic acid, and porphyrins, expanding its potential for future studies and biosensing applications.^[7]^

HSA is highly water-soluble and has an isoelectric point around *pH* 5, resulting in a net negative surface charge within the experimental *pH* range of 5.5–7. It displays a sharp UV-Vis absorption peak at approximately 280 nm (Figure S12). This negative charge supports strong immobilization on positively charged surfaces, such as amine-terminated linkers (e.g., cysteamine or APTMS). The abundance and uniform distribution of surface-active amino acid residues (Figure S11C–F) enable a variety of surface chemical reactions. Surface-exposed cysteine residues offer a notable advantage, allowing direct covalent binding to coinage and noble metals (Au, Ag, Cu) via metal-sulfur (M–S) bonds, eliminating the need for intermediate linkers. Furthermore, the presence of both acidic and basic amino acids supports efficient EDC-mediated coupling reactions,^[8]^ forming inter-protein amide bonds crucial for multilayer assembly. The asymmetric molecular structure of HSA (PDB: 1BM0)^[9]^ and the even distribution of its active residues allows for multiple immobilization orientations. Crystal structure data indicate that HSA’s longest dimensions span ~7.8 nm along the x- and y-axes (Figure S11A) and ~3.8 nm along the z-axis (Figure S11B). Interestingly, the observed layer thickness, regardless of substrate or the number of layers, was consistently ~4 nm, as measured by ellipsometry and AFM, except for the first layer on Au (~3 nm). This suggests a preferred orientation where the x–y plane serves as the thermodynamically favorable binding surface. Such orientation enables optimal interaction with both the substrate and adjacent protein molecules, supporting efficient multilayer formation.

**S4. HSA Thin Film Preparation**

**S4.1. HSA-SAM Preparation**

Self-assembled monolayers (SAMs) of HSA were prepared on three different substrates: p^++^-Si/SiO_x_, gold (Si/Au), and MpD with a bottom Au electrode. On the Si/SiO_x_ substrate, HSA was electrostatically immobilized onto a positively charged, amine-terminated 3-aminopropyltrimethoxysilane (APTMS, Sigma) linker layer, which was attached to a <1 nm regrown silicon oxide layer on a highly doped (>10^20^ cm^-3^) Si wafer. The detailed procedures for preparing the substrate and applying the APTMS layer are described in recent work.^[1]^ The APTMS-coated p^++^-Si/SiO_x_ substrate was incubated in a standard HSA solution (2 mg/mL in 20 mM phosphate buffer with 150 mM NaCl, pH 5.5) for 6 hours. After incubation, the electrostatically bound protein layer (Figure S13) was thoroughly rinsed with Milli-Q water and dried under nitrogen gas.

The same immobilization method was employed for HSA on both the Si/Au and MpD substrates, for MpD, HSA binds directly to the bottom Au electrode (via Au-S) without any linker layer. First, the substrates were cleaned by sequential sonication in acetone, isopropanol (IPA), and Milli-Q water for 3 minutes each. This was followed by a 20–25 second treatment with hot base piranha solution (H_2_O: H_2_O_2_: NH_3_ = 5: 1: 1 v/v) at 80 °C to activate the Au surface for protein immobilization. After treatment, the substrates were thoroughly rinsed with Milli-Q water and dried with nitrogen gas. The activated surfaces were immediately incubated in the standard HSA solution (as described above) for 12 hours. Following incubation, the HSA-modified substrates were rinsed with Milli-Q water and dried under a flow of N_2_ gas.

**S4.2. Preparation of HSA Multilayers**

HSA multilayers were prepared on various substrates using EDC coupling, as described in prior studies.^[1]^ The procedure began with the activation of the HSA monolayer by incubating it in a freshly prepared EDC solution (20 mg/mL in 20 mM phosphate buffer containing 150 mM NaCl, pH 5.5) for 30 minutes. Following incubation, the substrate was rinsed with phosphate buffer to remove any unreacted EDC from the protein surface. Care was taken to remove the excess buffer from the substrate’s bottom side while ensuring that the EDC-treated protein surface remained slightly hydrated with trace amounts of the buffer. Complete drying of the EDC-activated surface was avoided, as this would result in the decomposition of activated carboxylate groups back to their unreactive carboxylate form. The buffer-wetted, EDC-activated protein surface was immediately treated with a standard HSA solution (2 mg/mL in 20 mM phosphate buffer with 150 mM NaCl, pH 5.5) and incubated for 6 hours. Following incubation, the substrate was rinsed with Milli-Q water and dried with nitrogen gas. The EDC activation and HSA incubation steps were repeated as needed to achieve the desired number of HSA layers. During the treatment with HSA solution, the activated carboxylate groups on the (immobilized) protein surface reacted with the amine groups of lysine residues exposed on adsorbate HSA molecules, forming covalent inter-protein amide linkages (see Figure S13). This procedure was highly reproducible, allowing precise control over HSA multilayer growth and thickness.

**S5. bR Thin Film Preparation**

In parallel, we conducted a study employing bacteriorhodopsin (bR) treated with octylthioglucoside (OTG), as described in detail in our preceding publications.^[1,10]^ The immobilization of bacteriorhodopsin (bR) was carried out in a bilayer fashion.^[1]^ Protocols for preparing single bilayers and multilayers on linker-coated p^++^-Si/SiO_x_ and Au substrates are provided in ref. ^[1]^. For bR multilayers in MpD, a slightly modified procedure was used as follows:

**S5.1. Substrate Cleaning**

MpD substrates were sonicated sequentially in acetone, isopropanol (IPA), and Milli-Q water for 3 minutes each. This was followed by a 20-25 sec treatment with hot base piranha (H_2_O: H_2_O_2_: NH_3_ =5:1:1 by v/v) solution (80 °C) to activate the surface. The substrates were then thoroughly rinsed with Milli-Q water and dried using nitrogen gas.

**S5.2. Deposition of Linker Layer**

The cleaned MpD substrates were incubated overnight in a solution of cysteamine hydrochloride (cys) (4 mg/mL) in a pH 7.4 phosphate buffer medium. Following the incubation process, the cys-treated MpD was extensively rinsed with Milli-Q water, followed by 30-sec bath sonication to remove unbound cysteamine, and dried with nitrogen gas.

**S5.3. bR Bilayer(s) Preparation**

The cys-modified MpD substrates were then incubated in a 4 *µ*M solution of octylthioglucoside (OTG)-solubilized bR in 10 mM phosphate buffer (PB) containing 0.1 M ammonium sulfate ((NH_4_)_2_SO_4_) at pH 6.4 for overnight. After incubation,^[1]^ the bR-modified MpD was rinsed with Milli-Q water and dried with nitrogen gas. This procedure resulted in the formation of a single bR bilayer on MpD. For the preparation of bR multilayers, the protocol described in ref. ^[1]^, was followed.

**S6. Procedure for the Top Electrode Deposition**

The protein (HSA or bR) modified bottom electrode was subjected to three different top electrode depositions as follows.

**S6.1. Si-Au Junctions**

For the Si-Au junctions, the top Au-pads were deposited on the protein layer immobilized on p^++^-Si/SiO_x_/APTMS substrates as described in a recent work.^[1]^

**S6.2. MpD Devices and Probe Connections**

The fabrication of micropore device (MpD) structures was carried out as detailed elsewhere.^[11]^ Each chip contained 16–56 micro-junction devices.^[11]^ A top view of the entire MpD chip and a cross-sectional schematic of a single MpD junction are presented in Figure S14.

The bottom electrode of each device, consisting of an exposed Au surface, was electrically isolated. Protein multilayers were selectively deposited onto the micropore areas (~10^-7^ cm^2^) of the Au surface, while the surrounding regions were insulated with alumina (see details in ref.^[11]^). The top electrode was formed by semi-indirect E-beam deposition of a 35 nm Pd layer, followed by a 25 nm Au protective coating, using an advanced evaporator system (Angstrom Evaporator), as detailed in ref.^[11]^. This top electrode served as the common (central) contact for all micro-junctions, to which the bias voltage was applied, while the bottom electrode was grounded (Figure S14A).

**S6.3. EGaIn-Cone-Based Top Electrode Landing and Measurement Setup**

HSA and bR protein layers were prepared on Au substrates as described in Sections S4 and S5. A home-built setup was used to prepare Au-EGaIn junctions. Freshly prepared EGaIn (from Sigma) micro-cones with a typical tip diameter of ~10^-4^ cm^2^ were formed using a conductive metallic cylindrical probe attached to a micromanipulator. The EGaIn micro-cone landed softly on the protein surface (under live optical microscope monitoring) to form the top electrode (see Figure S8).

The experimental setup was mounted on a vibration-isolated table equipped with a vertical microscope for visualizing the protein-EGaIn contact and precisely measuring the contact area. The EGaIn top contact was connected either to an AC impedance analyzer (*Zurich Instruments MFIA*) or to a DC source meter (*Agilent B2911A*) with fA-level noise sensitivity. The substrate-attached protein layer was properly grounded during all measurements; for experimental parameters, see details in Section S2.1*.*

**S7. Quality and Thickness of HSA/bR Films**

The thickness of the protein layers was primarily monitored by ellipsometry (see Table S3). For the Si-Au and Au-EGaIn junctions, ellipsometry measurements were performed directly on the protein-coated bottom electrodes prior to top electrode deposition. Due to the limited size of MpD junctions, direct ellipsometric characterization at the active area, was not feasible. Instead, protein layer thicknesses for these junctions were measured on simultaneously prepared protein films on Au substrates, identical to those used in micropore fabrication. Protein layer growth was found to be uniform across different substrates, with a thickness of ~4 nm per layer for HSA and ~8nm per bilayer for bR.

Up to four successive HSA layers were studied. For the Si-Au junctions, transport behavior was measured for mono-, bi-, tri-, and tetra-layered HSA films. However, for junctions on gold substrates (Au-EGaIn and MpD), only bi-, tri-, and tetra-layer junctions were characterized. HSA monolayers on gold substrates resulted in electrically shorted junctions, which we attribute to nanometer-scale inter-protein voids.^[8]^ For bR, junctions with one, two, and three bilayers^[1]^ (simply considered as mono, bi, tri-layers) were studied in both the Si-Au and Au-EGaIn configurations. As with HSA, all single bR bilayer junctions using the micropore configuration were shorted. Consequently, double-, triple-, and quadruple-bilayer (tetralayer) junctions were analyzed to obtain three reliable data points for length-dependent studies.

The quality of the HSA layers was assessed using tapping-mode AFM imaging (*Nanoscope V Multimode* AFM setup) under ambient conditions, as described previously.^[1,12]^ The quality and AFM characterization of bR bilayers have also been reported earlier.^[1,10]^ AFM images revealed dense and uniform coverage of protein molecules across the substrates, with comparable RMS roughness on both p^++^-Si/SiO_x_/APTMS and Au surfaces, including within the micropores (Figure S15). To further validate thickness measurements, AFM scratching experiments were performed^[1,11,12]^ on HSA bilayers on Au, HSA trilayers on MpD, and HSA tetralayers on APTMS-coated p^++^-Si/SiO_x_ (Figure S16). The protein thickness values obtained from these experiments were in close agreement with ellipsometry data (see Table S3) for both HSA and bR^.^ AFM-scratching-based layer thickness measurements of various bR layers were studied in detail in our recent work.^[1,11]^

Additionally, PM-IRRAS (polarization modulation-infrared reflection-absorption spectroscopy) of HSA trilayers revealed characteristic amide I and amide II bands (Figure S12A), consistent with known protein spectra such as those of bR,^[1]^ streptavidin,^[12]^ azurin,^[13]^ and others.^[14]^ Detailed procedures for these analyses are provided in our previous work.^[12]^

**S8. Quality Assessment of Protein-Based Devices via Impedance Analysis**

Impedance-derived phase plots provide valuable insight into the formation of high-quality protein junctions. As discussed in Section 2.1.2 of the main text, under an applied AC field, the high-frequency regime (>10 kHz) predominantly drives AC current through the capacitive (*C_P_*) component of protein junctions (see Scheme 1 in the main text). Consequently, a characteristic AC phase lag approaching 90° is expected due to the partial capacitive nature of the junction, such a signature was clearly observed in the experimental protein junctions (Figure S17).

Under these conditions, protein junctions behave equivalent to a capacitor, where the sandwiched protein layers act as a dielectric medium that electrically isolates the two terminal electrodes. This configuration gives direct evidence for the prevention of filamentous growth or direct conduction (shorted) between the terminal electrodes, which is particularly crucial for MpD configuration. As a result, impedance-derived phase plots are valuable tools for identifying transport-active junctions while excluding those that exhibit shorting or partial shorting, as discussed in our current work.^[11]^

**S9.** **Inverse Capacitance-HSA/bR Thickness Relation**

The capacitance of a parallel plate capacitor depends inversely on the dielectric separation between the plates (in our case, the protein layer thickness). Therefore, a plot of capacitance (*C*_P_) versus inverse film thickness (1/*d*) is expected to be linear,^[1,15]^ as shown in Figure S18. The linearity is maintained nicely for Si-Au junctions of HSA layers (shown here) and bR layers (see ref. ^[1]^). However, MpD junctions (for both HSA and bR) do not exhibit significant *C*_P_ variation with 1/*d* (Figures S18C, S18D), likely due to the presence of alumina (Al_2_O_3_) insulator that constrains the geometry of the micropore devices (see in SI, Section S6.2, and Figure S14).^[11]^

A single MpD chip integrates 16 to 56 devices,^[11]^ each featuring a top (Pd/Au-evaporated) contact area of approximately 10,000 *µ*m² (see Figure S14). Over 99% of the device area is composed of highly insulating ALD-deposited alumina (Al_2_O_3_, ~20 nm thick), which separates the top and bottom metal electrodes. A small central micropore (~0.2% of the total area), measuring approximately 20 *µ*m^2^ with a depth of ~20 nm, serves as the active site.^[11]^ The build-up protein layers (HSA or bR) specifically within this micropore cover only a negligible fraction of the total device area. Alumina, with a dielectric constant of ~10,^[16]^ has a significantly higher permittivity than the protein layers (~3–4).^[17,18]^ Consequently, the overall device capacitance is dominated solely by the alumina, and variations in the protein layer thickness within the micropore have a minimal impact on the measured capacitance. This behavior was observed consistently in both HSA and bR junctions within the MpD configuration (see Figures 18C and D). In contrast, the impedance-derived capacitance of Si-Au and Au-EGaIn junctions is closely correlated with protein layer thickness, as the protein spans the entire device area (unlike MpD) in these configurations. For these junctions, the estimated dielectric constant was ~2, relatively low, possibly due to the absence of cofactors in HSA, and lower than what was observed in bR-based junctions (~5).^[1]^

**S10. Monitoring Protein Layer Thickness**

The study on the length dependence of ETp necessitates precise control over the layer thickness in the experimental protein junctions. As detailed above (see Section S7), we monitored protein layer thickness using direct methods for Si-Au and Au-EGaIn junctions and indirect methods for MpD, employing ellipsometry and occasionally AFM scratching experiments. The detailed experimental methodologies have been previously described.^[1,12]^

**S10.1. Ellipsometry**

The protein layer thickness was determined using the *Cauchy* model by fitting the psi-delta data over the wavelength range of 350–1000 nm.^[1,12]^ This analysis was conducted with the software of the *Woollam M2000 V* ellipsometer.

**S10.2. AFM Scratching**

High-force AFM contact mode imaging (applied force: 160–200 nN)^[1,8,12,19]^ was used to scratch protein surfaces. Scratching experiments were performed on bilayer, trilayer, and tetralayer HSA deposited on Au substrates, MpD, and Si/APTMS, respectively. *Gwyddion 2.63* software was used for AFM image processing, including roughness calculation and image presentation.

**S11. Junction Resistance from *J-V* Slope**

The junction current was quantified in terms of current density, normalized by the geometric contact area of the protein junctions as described in the main text. The reciprocal of the averaged *J-V* curve (within the linear portion near 0 V applied bias) for various protein layers provided the area-normalized junction resistance (in Ω·cm^2^) for each protein layer within a given device configuration from Figure S2, S3. In this study, we used the geometric junction area for area normalization. While this approach serves as a rough approximation for large-area,^[20]^ mechanically contacted junctions, such as Si–Au and Au–EGaIn junctions, it may offer improved accuracy for relatively smaller, precisely controlled junctions, particularly those involving evaporated top electrodes composed of MpD. Additionally, the same methodology was employed to determine the junction resistance of bare devices (electrically shorted configurations without any protein layer) across the Si-Au, Au-EGaIn, and MpD configurations.

**S12. Protein Structural Analysis**

Using the specialized PDB visualization and analysis tools *PyMOL* and *JSmol*, we examined the structures of our experimental proteins. For bacteriorhodopsin (bR), we selected the trimeric structure with PDB ID: 1BRR, while for human serum albumin (HSA), we used the monomeric structure with PDB ID: 1BM0.

**Figure S1.** Nyquist and Kramers-Kronig (KK) plots for different HSA and bR junctions (labels shown top-left in each subplot). Columns 1 and 3: Nyquist plots; Columns 2 and 4: corresponding KK plots. In Nyquist plots, black circles = experimental data, red lines = fitted curves. In KK plots, green squares = %*Z*_Error_ (*Z*_Re_), blue circles = %*Z*_Error_ (*Z*_Im_).

**Figure S2**. (**A-C**) Plots of averaged *J-V* curves for devices without proteins (shorted ones) in each of the three contacting configurations, as indicated in the respective figure legends. The reciprocal of the *J-V* slope yields the DC circuit resistance (DC-*R*_S_) for each device configuration.

**Figure S3**. Overlay of average *J-V* (20-30) traces of different protein junctions; the different HSA junctions are shown in the **TOP** row with (**A**) Si-Au, (**B**) Au-EGaIn, and (**C**) Au-Pd (MpD) device configurations. The **BOTTOM** row shows the same, but for bR junctions; in (**D**) Si-Au, (**E**) Au-EGaIn, and (**F**) MpD configuration.

**Figure S4**. Overlay of averaged protein resistance values (*R*_P_), derived from impedance fitting (AC) and the total junction resistance (*R*_T_), from the direct current (DC) measurements, for various HSA and bR junctions. HSA junctions in configurations of (**A**) Si-Au and (**C**) Au-Pd (MpD) and for bR junctions in configurations of (**B**) Si-Au and (**D**) MpD. Red dotted circles show the *R*_S_ values of the respective junctions, derived separately from AC and DC measurements.

**Figure S5**. **(A)** Schematic representation of the equivalent circuit model used to fit impedance data for protein junctions. **(B-E)** Representative Nyquist plots for various protein junctions; **(B-D)** for **HSA** and **(E)** for **bR**, **(B)** in Si-Au configuration with different layers as indicated in the figure legends, **(C)** of HSA bilayer in Au-EGaIn configuration, **(D)** and **(E)** for HSA and bR in the MpD configuration; **(D1, E1)** a bilayer, **(D2, E2)** a trilayer, and **(D3, E3)** a tetralayer. The colored dots represent experimental data points, while the black solid line shows the fit to the equivalent circuit (**A**), demonstrating a good fitting quality with χ^2^ ≈ 10^-3^.

**Figure S6.** Top views of surface-exposed polar/charged residues of **bR** trimer and **HSA** monomer (gold-surface-color) that can interact with the contact surface. The view is chosen so that we look at the protein surfaces that are assumed to bind directly to the substrate, via surface-exposed cysteines for HSA and electrostatically for bR. (**A**) White spaces within the bR trimer structure indicate cavities or hydrophobic regions, while magenta-colored regions highlight visible secondary structures through these cavities. Structures were analyzed using *Jsmol*: (**A**) bR-trimer (PDB: **1BRR**) and (**B**) HSA-monomer (PDB: **1BM0**).

**Figure S7**. *ln* (*R*_P_) versus thickness plots for the different protein junctions with mostly comparable protein thicknesses *(d ~*8 nm) with HSA or bR, along with *d* ~16 nm bR in MpD configuration, relative to the resistance of a bare device (*d* = 0).

| Table S1 | | |
| --- | --- | --- |
| Junctions | Thickness of protein layer (nm) | *β* (nm^-1^) |
| Au/**HSA**/Pd-Au | 7.25 ± 0.75 | 1.10 ± 0.10 |
| Au/**HSA**/EGaIn | 7.50 ± 0.50 | 2.30 ± 0.15 |
| Si/SiO_x_/APTMS/**HSA**/Au-pad | 7.20 ± 0.40 | 1.80 ± 0.10 |
| Au/Cys/**bR**/Pd-Au | 16.0 ± 1.00 | 0.60 ± 0.05 |
| Au/Cys/**bR**/EGaIn | 7.80 ± 0.40 | 1.60 ± 0.05 |
| Si/SiO_x_/APTMS/**bR**/Au-pad | 9.00 ± 1.00 | 0.95 ± 0.10 |

**Table S1**. Distance decay parameters *(β* values) were obtained from the slope of *ln* (*R*_P_) vs. thickness (from Figure S7, shown with fitted dotted/dashed lines).

**Figure S8**. (**A**) Schematic representation of the Au/protein/EGaIn^cone^ (Au-EGaIn) junction used for transport measurements. (**B**) Actual photograph of the junction, showing a side view of the EGaIn-protein contact, with the interface highlighted by a *yellow* dotted line.

**Figure S9.** The representative of -*Z*_Im_ versus frequency plots for various protein junctions, as indicated in each figure. Colored dots represent the actual data, while the solid black line shows the fitted data. Each plot features a single peak, indicating a single dielectric process. The gray shaded region (96% confidence limit) represents the frequency range where the actual dielectric process occurs. The peak of the plot indicates the time constant of the process.

| Table S2: Si-Au junction: Impedance derived *R*_S_  vs *DC-R*_S_ | |
| --- | --- |
| Junction | *R_S_* [Ω.cm^2^] |
| p^++^-Si/SiO_x_ (1nm)/Au (shorted) | 7.8 × 10^-2^ |
| p^++^-Si/SiO_x_ (1nm)/linker(0.5nm)/Au | 4.6 × 10^-2^ |
| p^++^-Si/SiO_x_ (1nm)/linker/HSA(11 nm)/Au | 5.5 × 10^-2^ |
| p^++^-Si/SiO_x_ (1nm)/linker/bR (9 nm)/Au | 5.2 × 10^-2^ |
| p^++^-Si/SiO_x_ (1nm)/linker/PAH-PAA (13 nm)/Au | 5.1 × 10^-2^ |
| p^++^-Si/SiO_x_ (2 nm ALD growth)/Au | 6.4 × 10^-2^ |
| p^++^-Si/SiO_x_ (2 nm ALD growth)/linker/HSA(3.5 nm)/Au | 4.0 × 10^-2^ |
| DC-*R*s (p^++^-Si/SiO_x_(1nm)/Au ) | ~5× 10^-2^ |

**Table S2**: Estimated *R*_S_ values for different Si-Au junctions from impedance fit, and the bottom ROW of this table represents the DC-*R*_S_ values of corresponding shorted junctions, estimated from the reciprocal of *J-V* (DC) plot. Here, ‘PAH-PAA’ is the layer-by-layer growth of successive polycationic and polyionic polymeric multilayers.

**Figure S10.** DC *J–V* and impedance responses of shorted devices without protein/linker layers. Columns: (1) Au-EGaIn, (2) MpD, (3) Si-Au junctions (as labeled). Rows: (1) *J–V* curves with DC-*R*_S_, (2) Nyquist plots, (3) *–Z*_Im_ vs. frequency, (4) phase plots. Colored circled dots = experimental data; solid black lines = fitted curves.

**Figure S11**. The crystal structure of human serum albumin (HSA), analyzed using *PyMOL*, is based on the Protein Data Bank (PDB) entry 1BM0 (monomer). (**A**) A quasi-3-D representation of the HSA monomer; the green shows the protein’s secondary and tertiary structure; the outermost part of the semi-transparent gray volume defines the protein’s outer boundary; the three principal dimensions are indicated by arrows. (**B**) The preferred surface orientation of the HSA monomer is the 'lying down' configuration. (**C**)-(**F**): Specific amino acid residues with potential activity are highlighted on the HSA structure; how many of each are there appears in parentheses: (**C**) Aspartic acid residues (35) in red, (**D**) Glutamic acid residues (62) in magenta, (**E**) Lysine residues (58) in orange and (**F**) Cysteine residues (35) in yellow.

**Figure S12**. (**A**) PM-IRRAS spectral signature of HSA trilayer (~11.5 nm) on Au-substrate over a full spectral range showing the typical amide I and II characteristics (shaded zone) as seen also for other solid-state protein layers (*see Section S7 in SI*). (**B**) A typical UV-Vis spectrum of a 2 mg/mL HSA solution in 20 mM phosphate buffer at pH 5.5 in 150 mM NaCl medium, collected from Nanodrop with a 1 mm path length.

**Figure S13**. Schematic representation of layer-by-layer HSA multilayer on (**A**) Au-substrate/MpD and (**B**) p^++^-Si/SiO_x_/APTMS, with the interface charges indicated. Net surface charges from carboxylate groups (aspartic and glutamic acids) on the protein envelope are shown for the first layer.

**Figure S14**. (**A**) Schematic representation of an MpD chip shows an array of 16 devices used for the measurements. The top electrode, a Pd-Au layer (yellow square), serves as the central common electrode, and separate bottom electrodes are used for biasing each device. The red squares highlight the protein layers within the micropores. (**B**) A cross-sectional schematic view of the complete MpD device, showing the stacked protein layers. The thickness (in nanometers) of all the device layers is marked by vertical black arrows. Additionally, a side view of the protein-covered part of the individual bottom electrodes (4 × 4 *µ*m^2^) is provided at the center of the micropore area (100 × 100 *µ*m^2^), as indicated by the red horizontal arrow in one of the two lateral directions.

| Table S3A: HSA Layer Thickness (nm) from Ellipsometry | | |
| --- | --- | --- |
| HSA layers | Si/SiO_x_/APTMS/**HSA** | Au/**HSA** |
| Monolayer | 3.40-4.00 | 2.80-3.50 |
| Bilayer | 6.80-7.60 | 6.50-8.00 |
| Trilayer | 10.5-11.5 | 10.8-12.0 |
| Tetralayer | 15.5-17.0 | 15.8-16.4 |

| Table S3B: bR Layer Thickness (nm) from Ellipsometry | | |
| --- | --- | --- |
| bR layers | Si/SiO_x_/APTMS/**bR** | Au/cys/**bR** |
| Monolayer | 8.20-9.40 | 7.60-8.10 |
| Bilayer | 15.0-17.0 | 15.0-17.0 |
| Trilayer | 24.0-30.0 | 22.0-27.0 |
| Tetralayer | -- | 32.0-35.0 |

**Table S3**. (**A**) Ellipsometry-derived thicknesses for HSA and (**B**) bR layers on both Si and Au substrates. The average thicknesses of the linker layers were: (APTMS) 0.5±0.1 nm, cysteamine (cys) 0.6±0.1 nm, and the SiO_X_ layer was 0.9±0.2 nm thick.

**Figure S15**. Tapping mode AFM topography images of HSA monolayers on different electrode (substrate) surfaces: **(A)** Au substrate, **(B)** micropore region of MpD, and **(C)** p^++^-Si/SiOx/APTMS. The corresponding RMS roughness values are 1.0 ± 0.05 nm, 1.2 ± 0.2 nm, and 1.2 ± 0.1 nm, respectively.

**Figure S16**. **Left column**: HSA bilayer on an Au substrate; **Middle column**: HSA trilayer on MpD; **Right column**: HSA tetralayer on Si/SiOx/APTMS. Panels **(A1, B1, C1)** show AFM topography images of scratched regions (marked by black squares), while panels **(A2, B2, C2)** present cumulative five-line profiles across the corresponding scratched areas. From these profiles, we estimate the thickness of the HSA multilayers: **7.5 ± 0.5 nm** for the bilayer, **11 ± 0.5 nm** for the trilayer, and **16 ± 1.0 nm** for the tetralayer. In panel **(B1)**, the scratched region (highlighted with a *dotted red* circle) is located within the center of the micropore area (~4 × 4 *µ*m²) of the MpD surface.

**Figure S17**. Bode plots (phase-shift vs. operating frequency) for protein junctions in the different device configurations. (**A-C**) HSA: (**A**) Si-Au, (**B**) Au-EGaIn, and (**C**) MpD. (**D**) is for the bR junctions in MpD configuration. In ref.^[1]^, the corresponding results for the Si-Au junction are given. Here, the colored dots represent the experimental data, while the black solid line shows the fit based on the equivalent circuit (S5A), with a fitting quality of χ^2^ ≈ 10^-3^.

**Figure S18. (A-C):** Plots of *C*_P_ vs. 1/*d* of different HSA junctions in **(A)** Si-Au, **(B)** Au-EGaIn, and **(C)** Au-Pd (MpD) configurations and **(D)** bR junctions in the MpD configuration. The different protein layers are labeled as L1, L2, L3, and L4, representing a monolayer, bilayer, trilayer, and tetralayer, respectively.

**References**

[1] S. Bera, J. A. Fereiro, S. K. Saxena, D. Chryssikos, K. Majhi, T. Bendikov, L. Sepunaru, D. Ehre, M. Tornow, I. Pecht, A. Vilan, M. Sheves, D. Cahen, *J. Am. Chem. Soc.* **2023**, *145*, 24820.

[2] J. Bisquert, F. Fabregat-Santiago, In *Dye-sensitized Solar Cells*, EPFL Press, **2010**.

[3] X. Chen, C. A. Nijhuis, *Advanced Electronic Materials* **2022**, *8*, 2100495.

[4] C. S. S. Sangeeth, A. Wan, C. A. Nijhuis, *J. Am. Chem. Soc.* **2014**, *136*, 11134.

[5] C. S. S. Sangeeth, A. Wan, C. A. Nijhuis, *Nanoscale* **2015**, *7*, 12061.

[6] Y. Xie, Z. Cao, W. Peng, Y. Li, *Advanced Materials Technologies.* **2025***,* e00674.

[7] S. M. Petrovic, M.-E. Barbinta-Patrascu, *Materials* **2023**, *16*, 7550.

[8] S. Bera, J. Kolay, P. Pramanik, A. Bhattacharyya, R. Mukhopadhyay, *Journal of Materials Chemistry C* **2019**, *7*, 9038.

[9] S. Sugio, A. Kashima, S. Mochizuki, M. Noda, K. Kobayashi, *Protein Engineering, Design and Selection* **1999**, *12*, 439.

[10] K. Garg, S. Raichlin, T. Bendikov, I. Pecht, M. Sheves, D. Cahen, *ACS Appl. Mater. Interfaces* **2018**, *10*, 41599.

[11] S. Bera, E. Mishuk, P. Li, S. Das, S. Keshet, S. Garusi, L. Tunik, E. Edri, Y. Selzer, I. Pecht, A. Vilan, M. Sheves, D. Cahen, *Hard-wired Solid-state Bioelectronic Micropore Devices: Permanent Metal-Protein-Metal Junction Proof-of-Concept*, ChemRxiv, **2025**.

[12] S. Bera, S. Govinda, J. A. Fereiro, I. Pecht, M. Sheves, D. Cahen, *Langmuir* **2023**, *39*, 1394.

[13] J. A. Fereiro, G. Porat, T. Bendikov, I. Pecht, M. Sheves, D. Cahen, *J. Am. Chem. Soc.* **2018**, *140*, 13317.

[14] D. Chryssikos, J. A. Fereiro, J. Rojas, S. Bera, D. Tüzün, E. Kounoupioti, R. N. Pereira, C. Pfeiffer, A. Khoshouei, H. Dietz, M. Sheves, D. Cahen, M. Tornow, *Advanced Functional Materials* **2024**, *34*, 2408110.

[15] C. S. Suchand Sangeeth, L. Jiang, C. A. Nijhuis, *RSC Advances* **2018**, *8*, 19939.

[16] H. Birey, *Journal of Applied Physics* **1978**, *49*, 2898.

[17] F. Bibi, M. Villain, C. Guillaume, B. Sorli, N. Gontard, *Sensors* **2016**, *16*, 1232.

[18] M. Amin, J. Küpper, *ChemistryOpen* **2020**, *9*, 691.

[19] S. Bera, J. Kolay, S. Banerjee, R. Mukhopadhyay, *Langmuir* **2017**, *33*, 1951.

[20] S. Mukhopadhyay, S. K. Karuppannan, C. Guo, J. A. Fereiro, A. Bergren, V. Mukundan, X. Qiu, O. E. C. Ocampo, X. Chen, R. C. Chiechi, R. McCreery, I. Pecht, M. Sheves, R. R. Pasula, S. Lim, C. A. Nijhuis, A. Vilan, D. Cahen, *iScience* **2020**, *23*.
